# Coral-seeding devices with fish-exclusion features reduce mortality on the Great Barrier Reef

**DOI:** 10.1038/s41598-024-64294-z

**Published:** 2024-06-10

**Authors:** T. N. Whitman, M. O. Hoogenboom, A. P. Negri, C. J. Randall

**Affiliations:** 1 Australian Institute of Marine Science (AIMS), Townsville, Australia; 2 College of Science and Engineering, James Cook University, Townsville, Australia; 3 AIMS@JCU, Townsville, Australia

**Keywords:** Coral ecology, Coral biology, Reef restoration, Fish ecology, Animal behaviour, Restoration ecology, Marine biology

## Abstract

Restoration methods that seed juvenile corals show promise as scalable interventions to promote population persistence through anthropogenic warming. However, challenges including predation by fishes can threaten coral survival. Coral-seeding devices with refugia from fishes offer potential solutions to limit predation-driven mortality. In an 8-month field study, we assessed the efficacy of such devices for increasing the survival of captive-reared *Acropora digitifera* (spat and microfragments) over control devices (featureless and caged). Devices with fish-exclusion features demonstrated a twofold increase in coral survival, while most corals seeded without protection suffered mortality within 48 h. Overall, spat faced more grazing and higher mortality compared to microfragments, and upward-facing corals were more vulnerable than side-facing corals. Grazing-induced mortality varied by site, with lower activity in locations abundant in mat-forming cyanobacteria or Scleractinian corals. Many scraping parrotfish were found feeding on or near the seeded corals; however, bites by *Scarus globiceps* explained the most site-related variation in grazing. Cyanobacteria may be preferred over corals as a nutritional resource for scraping parrotfish—advancing our understanding of their foraging ecology. Incorporating side-facing refugia in seeding devices and deploying to sites with nutrient-rich food sources for fish are potential strategies to enhance coral survival in restoration programs.

## Introduction

Anthropogenic warming has increased the frequency of severe disturbances on reefs. As a result, coral cover, coral population sizes and colony abundances have declined, reducing population-level fecundity and gamete-fertilization success, and thereby decreasing the number of coral propagules available for reef replenishment^1,2^. The successful dispersal and recruitment of coral propagules (via larval attachment, metamorphosis, and growth to maturity) plays a critical role in restoring coral populations after a disturbance^4,5^. However, mortality can be extremely high for recruiting corals, particularly on degraded reefs, and a minimum of 9 to 12 years free of major environmental perturbations is often required for reef communities to reach pre-disturbance levels of coral cover and diversity^6–8^. As corals continue to be impacted by increasingly frequent and acute disturbances combined with chronic local stressors, restoration initiatives should urgently prioritize techniques that can minimize critical bottlenecks to coral recruitment, thus accelerating natural recovery potential on degraded reefs.

Restoration scientists have tested several approaches to accelerate reef replenishment via assisted coral recruitment, including methods that optimize large-scale production and deployment of sexually produced coral larvae, spat or fragments^9,10^. Here, we define “coral seeding” as an intervention that harnesses the natural production of coral larvae from mass-spawning events, optimizes larval settlement ex situ, then deploys or ‘seeds’ settled spat (< 10 mm in size) back to reefs on artificial deployment devices^11–13^. This also includes seeding of small fragments (< 100 mm^2^, cut from mature-adult colonies; hereafter ‘microfragments’) that have the potential to boost growth rates and reduce time to reach size-escape thresholds from mortality and reproductive maturity^14–19^. Investigations into the feasibility of coral seeding as a tool for reef restoration are currently underway; however, research is needed to develop methods that overcome high coral mortality on devices with seeded spat, with less than 30% survival after one year being the norm^11,20–24^.

High mortality of juvenile corals can result from both direct and indirect ecological (biological and physical) interactions^3,25,26^. One frequently reported cause of post-seeding mortality is grazing dislodgement by herbivores and corallivores. For example, biting (direct or indirect) by fishes can remove or dislodge small, vulnerable corals from exposed surfaces^27–30^ and extreme, targeted grazing by territorial fishes can occur^31,32^. In response, the use of physical barriers (i.e., cages and metal spikes) to limit fish access and protect seeded corals has been explored^27,28^. Physical barriers can reduce grazing mortality but their use beyond experimental research has been intensely debated^33–35^. In summary, physical interventions are costly, time consuming, and not feasible to deploy over large areas—limiting their use in reef-scale restoration.

Excluding fishes clearly reduces dislodgement of vulnerable corals and increases survival, and the positive result of exclusion is evident early (i.e., within 8 days) and persists at least a year post-seeding^11,22,23,29,30^. However, the complete exclusion of fish can also increase crustose, foliose and turf algal biomass, increasing competitive interactions, partial mortality and disease susceptibility for young corals^36–41^. The environmental grazing pressure**—**measured as total surface area of fish bites relative to the cover of grazable substrate per year**—**can vary significantly by fish species, size and feeding style. For example, abundant grazers like parrotfish (Labridae, Scarini) can be categorized based on two feeding styles: “scraper”, where bites remove substrate surface material only, and “excavator”, where bites remove substantial chunks of substrate and substrate material, and this in turn dictates the ecological function of fish on reefs^42–46^. Yet functional roles of grazing fishes vary markedly due to reef hydrodynamics, structural complexity, and sediment characteristics^34,47,48^. Knowledge of these characteristics at the site level is important for understanding the interactions between grazing fishes and seeded corals because coral growth will also vary under such conditions^49,50^. Therefore, ecological assessments of coral, fish and the biological and physical habitat are required to tailor seeding techniques to the receiving environment for effective restoration.

Although research has progressed to incorporate protective features (e.g., protrusions, indentations, and microrefugia) into the design of seeding devices^11,13,23,24,30^, device designs are yet to be optimized. Consequently, the effects of fish-exclusion features across space and time are likely context specific and warrant further investigation. Furthermore, no direct comparison of early survival following deployment has been made between aquaria-reared coral spat and microfragments of the same species, and understanding coral performance across life stages can help guide restoration decisions. To address these knowledge gaps, we tested the performance of coral seeding devices with fish-exclusion features against featureless control devices and alongside fully-caged controls. From this, we resolve four research questions: (1) does the survival of seeded corals differ by device treatment?; (2) do grazing attempts on corals differ among device treatments?; (3) are patterns of survival and grazing consistent across seeding sites?; and (4) do the ecological characteristics of the seeding site influence the observed survival and grazing patterns? The data from this experiment provide important insights into the complex ecological relationships that influence early coral survival. The information can be applied to coral-seeding methods being developed as a part of the global restoration toolkit.

## Methods

### Coral spawning, settlement, and grow out

Coral colonies of the species *Acropora digitifera* (Dana, 1846) were collected (permit G21/38,062.1 issued by the Great Barrier Reef Marine Park Authority [GBRMPA]) from Davies Reef (central, midshelf Great Barrier Reef [GBR]) prior to the 2021 Autumn (February to March) coral-spawning event. Following collection, colonies were transported to outdoor, flow-through seawater aquaria (average light intensity 74 μmol photons m^−1^ s^−1^ and temperature 27–28 °C) in the National Sea Simulator at the Australian Institute of Marine Science (AIMS, Townsville, Queensland) and maintained until spawning. The timing of spawning and the numbers of colonies that contributed to mass cultures are reported in Supplementary Table S1. Gamete bundles were collected, separated, washed, and fertilized as described in Pollock et al. 2017^51^*.* Embryos were then transferred to 500 L larval rearing tanks at a stocking density of ~ 0.5–1 larva mL^−1^. Culture tanks received flowthrough 0.4 μm filtered seawater (FSW) at 27.5–28.0 °C and gentle aeration began ~ 16 h post fertilization. Larvae remained in rearing tanks and once they reached settlement competency (~ 7–12 d, as determined by daily competency assays) they were settled *en masse* on coral rearing plugs.

Coral larvae were settled on conditioned aragonite plugs that hosted a mixed community of crustose coralline algae (CCA; e.g., *Porolithon*, *Lithophyllum*, *Titanoderma;* ; see Supplementary Material Sect. 1.1) and biofilms to induce settlement. Aragonite coral “frag plugs” (20-mm Ø; Ocean Wonders) were used as coral larval settlement substrates. Clean frag plugs were placed in well-conditioned (mixed community of CCA and biofilms), semi-recirculating indoor aquaria (280 L, flow of 5 L min^-1^, average Photosynthetic Active Radiation [PAR] of 160 μmol m^-2^ s^-1^) for 2-months prior to settlement. For settlement, 167 plugs were placed into clean, polyvinyl chloride (PVC) trays and trays were distributed to 50 L acrylic tanks (1 holding tray per tank, 12 trays, and 12 tanks in total). Approximately 3500 larvae (~ 20 larvae per plug) were added per tank and allowed to settle over 72 h. Settlement success on individual plugs was then assessed under a dissecting microscope and additional larvae (up to 1500) were added to tanks with low settlement. The maximum number of larvae added to tanks during settlement was 5000. Approximately 9 larvae settled per plug (range of 0 to 46 spat per plug), totaling ~ 18000 individual spat on 2000 plugs.

Settled spat were maintained in a semi-recirculating indoor aquarium (280 L, flow of 5 L min^−1^) under a daily feeding regime (unenriched *Artemia* [1 nauplius mL^-1^], mixed microalgae [2000 cells mL^−1^; *Nannochloropsis oceania*, *Isochrysis* sp*.*, *Chaetoceros muelleri*, *Dunaliella* sp., *Proteomonas sulcate*], and enriched Rotifers [0.5 nauplii mL^−1^; *Brachionus plicatilis,* 60–180 micron; SELCO^©^ S.parkle]) and a consistent light profile (PAR, initial 20 μmol m^−2^ s^−1^ and increasing ~ 14 μmol m^−2^ s^−1^ weekly, with a maximum of 160 μmol m^−2^ s^−1^ by deployment). PAR was maintained at a low level to control the growth of benthic organisms that compete with spat, and to mimic the low-light conditions found in crevices where spat tend to preferentially settle^29,36^. Fragments (> 10-cm branch length) of adult broodstock were placed in the aquarium with spat plugs to promote uptake of symbiotic zooxanthellae, Symbiodiniaceae. The aquarium was cleaned weekly, and plug trays were rotated and repositioned fortnightly to reduce the effect of any within-tank environmental variability on spat growth. After 4 months, 260 plugs were haphazardly distributed among 130 seeding devices for deployment, resulting in 2 spat plugs per device, one upward facing and one side facing. Each plug had between 1 and 3 discrete spat that were ~ 5–10 polyps and < 8 mm (Ø) in size.

### Coral microfragmentation

Microfragments were generated from 5 adult broodstock colonies 3 months post spawning and 1 month prior to deployment. Colonies were first chiseled into large fragments (~ 10 cm length), then cut into uniform microfragments (8 × 8 mm, Gryphon Diamond Band Saw). Only branches with mature polyps (i.e., upward facing in the center of the colony) were chosen for microfragmentation^9^. Branches were first cross-sectioned; the upper facing side was then cut into microfragments. The microfragments were glued (Gorilla glue) to the center of clean (bleached and autoclaved) aragonite coral plugs (20 mm Ø) and maintained in aquaria for approximately 1 month for grow out. Microfragments (*n* = 692) on plugs were then distributed to 346 seeding devices (2 microfragments per device) for deployment. Individuals were evenly distributed among device treatments and orientation by genotype, and the devices were evenly seeded to the experimental sites.

### Seeding device treatments and deployments

**Figure 1:**
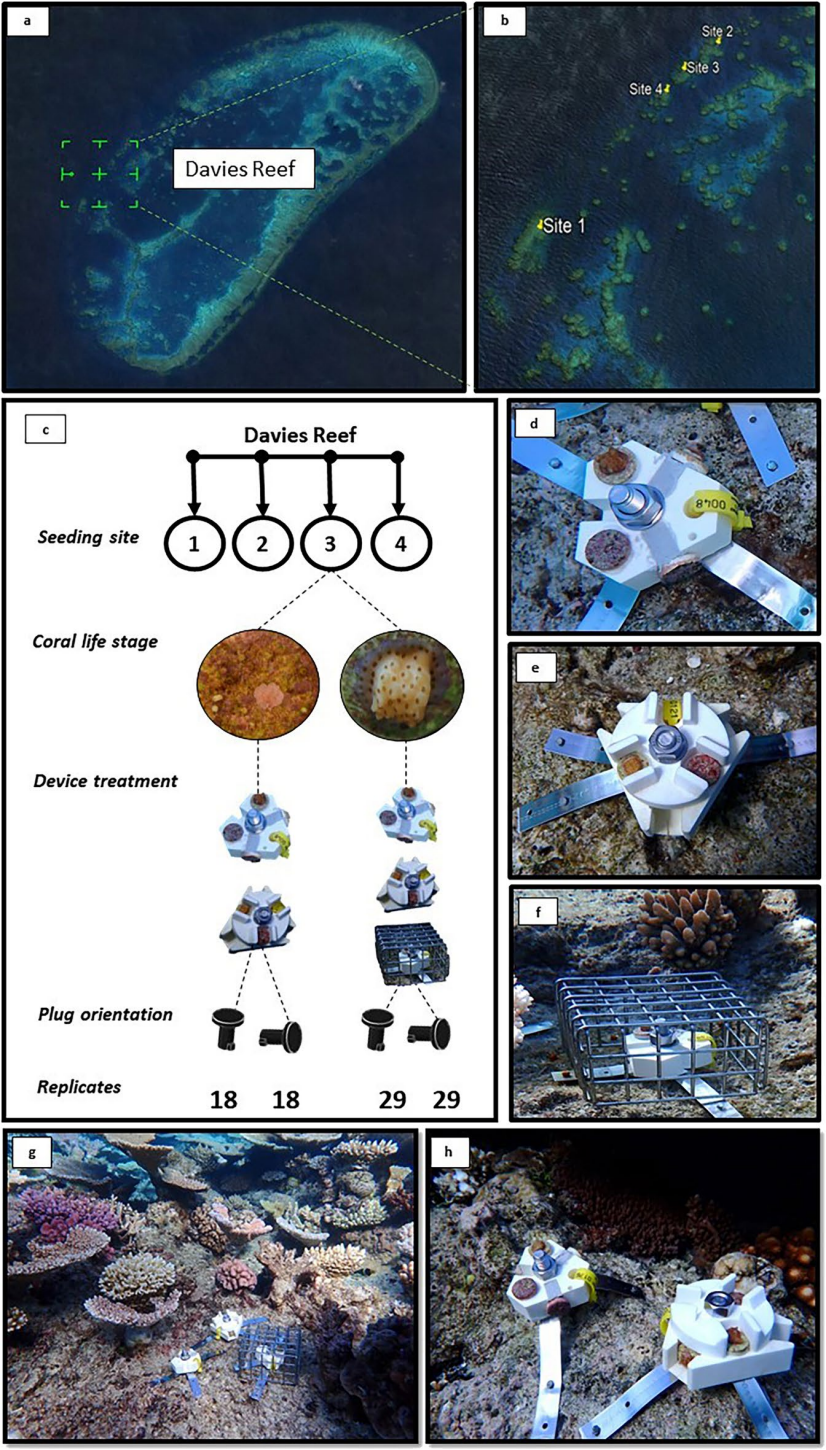
Site selection and design plan for the coral-seeding experiment. A total of 346 seeding devices were deployed to Davies Reef (**a**; Google Earth Image © 2023 Maxar Technologies) and fixed to the reef substrate at 4 sites (**b**; Google Earth Image © 2023 Maxar Technologies; see supplementary Tabe S2 for coordinates). The experimental design (**c**) included 2 coral life stages, 3 device treatments, and 2 plug orientations, with replication among categories. The device treatments (**d**-**f**) included featureless control devices (+ control; **d**), devices with fish-exclusion features (experimental treatment; **e**) and caged control devices (- control; f). Devices (*n* = 76–100) were paired based on device treatment and coral life stage and deployed to 0.25 m^2^ plots (*n* = 31–41; g-h) at each site (*n* = 4).

**Figure 2:**
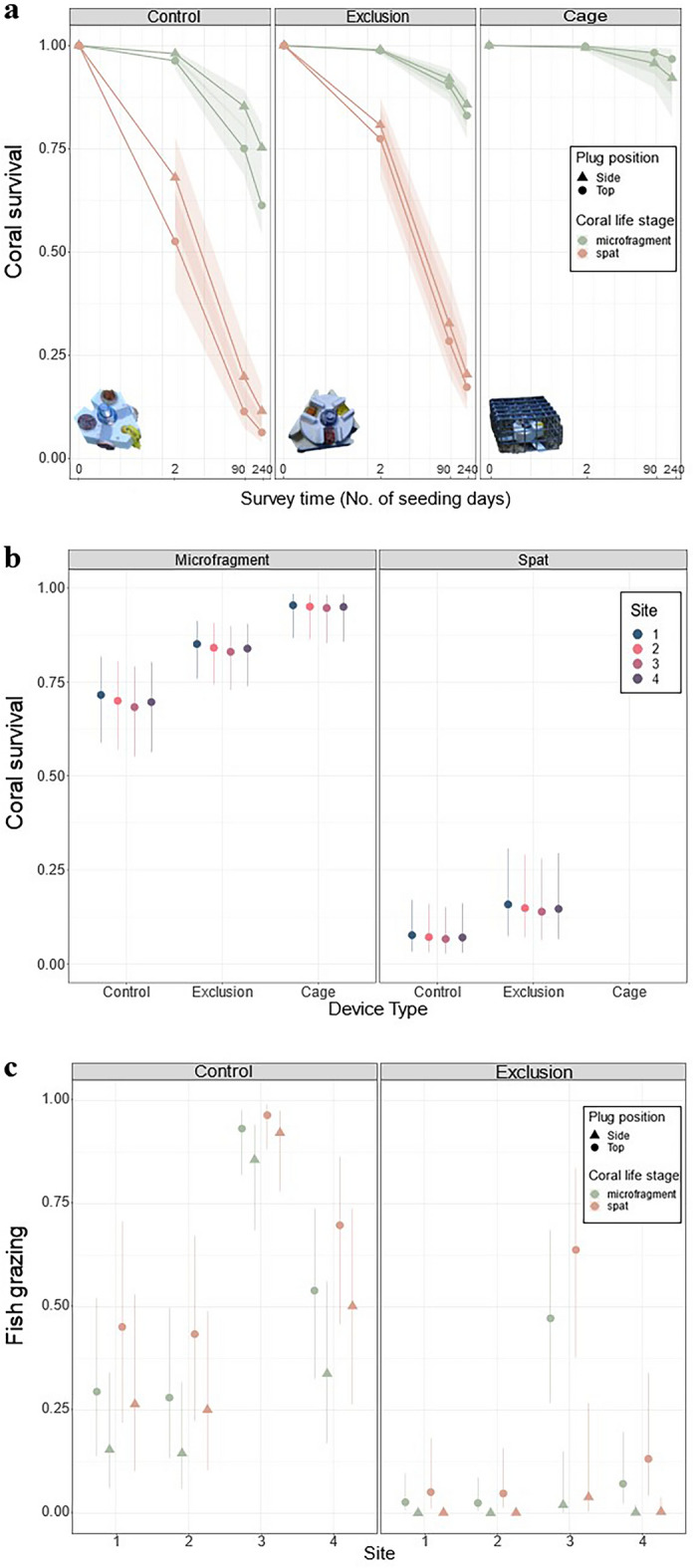
Average coral survival and grazing on seeded *Acropora digitifera*. (**a**) Coral survival through time by device treatment, coral orientation, and coral life stage (*n*_obs_ = 2456, *n*_groups_ = 4; see Supplementary Table S4). (**b**) Coral survival at 240 d separated by site, device type, and life stage (*n*_obs_ = 614, *n*_groups_ = 2). (**c**) Grazing by fish on seeded corals at 2 d allocated by site, device treatment, orientation, and life stage (*n*_obs_ = 380, *n*_groups_ = 149; Table S5). Points represent the modelled estimated marginal means with 95% confidence intervals. Note that the x-axis in (**a**) is on a log scale.

Reared spat and microfragments were deployed to Davies Reef (for site locations see Fig. 1a and Supplementary Table S2) in August 2021 and placed in 3 experimental treatments defined by the device type (Fig. 1d–f). Each device had nominally different levels of grazing exclusion and coral protection: (1) a featureless device acted as a positive control (open to grazing; Fig. 1d); (2) a caged featureless device acted as a negative control (complete exclusion of grazing by large fishes; Fig. 1f); and (3) a device with engineered fish-exclusion features (i.e., protrusions) acted as the experimental device (device of interest; Fig. 1e). The experimental device was designed at AIMS as a part of the Reef Restoration and Adaptation Program (RRAP) and represents the first design to support field deployment of juvenile corals grown on standard 20-mm (Ø) coral frag plugs. The devices were made of 95% alumina ceramic, which was selected for its chemical and physical stability (e.g., high specific gravity [3.8], hardness [9.0 Mohs]), and manufactured in the People’s Republic of China (Shanghai Gongtao Ceramics CO., Ltd. PRC). The device design provided the option for controlled spatial positioning (i.e., in relation to the seafloor) and modular components where the device could be customized for specific experiments; in this case, we selected modules with and without fish exclusion features.

Within each experimental device, there were 6 positions for coral plugs spread among 2 orientations: top- and side-facing. In the side orientation, the fish exclusion device had 12 mm protrusions on two sides, with open access for grazing from the top and no access from the bottom (Fig. 1e and h). The top orientation had 12-mm protrusions on two sides, a 10-mm protrusion at the inner edge and no protection on the outer edge (Fig. 1e and h). The minor differences in protection across plug orientations in the exclusion device were unavoidable due to assembly requirements but didn't appear to affect fish behavior (see results). The cage for the negative control was made from stainless steel metal fencing with a 25 × 25 mm grid (Fig. 1f). Devices were assembled and attached to the reef flat using a combination of metal strapping, bolts, nails, and cable ties, which were consistent among treatments (Fig. 1d–g). The central bolt used for device assembly provided an extra form of protection (i.e., physical barrier to large fish) to the inner edge of the coral plug on all devices (Fig. 1d–g). The devices were deployed onto 4 replicate sites at Davies Reef (Fig. 1a,b; Table S2) under GBRMPA permit G21/45,348.1 and were removed after 8 months.

The field deployment followed a hierarchical design (Fig. 1c) and included: sites (4, categorical, random, and fixed), coral life stage (2, categorical, fixed), device treatment (2 or 3, categorical, fixed), plug orientation (2, categorical, fixed) and replicates for each category. Sites (> 100 m apart) were located on the reef flat in 3m depth at high tide. Sites were selected based on their similarities in benthic community composition, their leeward location, and the presence of adult *A. digitifera* colonies. Within each site, devices (*n* = 76–100) were paired based on device treatment and coral life stage and deployed to 0.25 m^2^ plots (*n* = 31–41; Fig. 1g,h). Plots were placed haphazardly within each site and spaced at least 2-m apart. Due to the limited number of plugs with sufficient coral spat at the time of deployment, the cage treatment was only tested with microfragments. Therefore, sites had plots with paired devices (featureless and exclusion, Fig. 1d-e and 1h) and plots with all 3 devices (Fig. 1d–g). Paired plots (*n* = 17–23 site^−1^) had spat and microfragment plugs (*n* = 2 corals life stage^−1^, n = 1 orientation^−1^, *n* = 4 device^−1^) while plots with 3 devices (*n* = 13–18) contained microfragments only (*n* = 1 orientation^−1^, *n* = 2 device^−1^). In total, 346 seeding devices with 98 spat and 692 microfragment plugs were deployed.

### Biological and environmental data collection

Devices were monitored in situ during 3 survey time points (2, 90, and 240 days) post deployment. Data collection included a suite of quantitative and qualitative assessments to determine the influence of biological (fish abundance and grazing, benthic composition) and environmental (reef hydrodynamics and sedimentation) drivers on coral growth and survival, each of which are detailed below. More details about the data collection for this experiment can be found in Supplementary Table S2.

### Coral survival and grazing assessments

Coral plugs were imaged (Olympus TG6) by divers during each survey and images were assessed to determine the presence or absence of grazing marks (at time 2 d only) and surviving corals (all time points) at the level of coral plug. Corals in caged treatments were not imaged at 2 d due to the difficulty in removing and resecuring cages, and it was assumed that no grazing had occurred; corals in caged treatments were imaged at all survey timepoints thereafter. Grazing (presence or absence) was not assessed with imagery in subsequent timepoints due to the difficulty in distinguishing old from new bite marks on the plugs. Only devices that held live corals and remained fixed to the substrate for the entirety of the experiment (63 devices with spat and 275 devices with microfragments) were used to infer survival and grazing. Following the experiment, high-quality images were taken of each plug to validate in situ coral survival based on visual surveys.

### Fish community surveys and surveillance

Fish communities were observed using in situ stationary point count surveys (78.5 m^2^ for 5 min, 3 replicates per site) by divers at 3 timepoints (2, 90, and 240 d). Fish of the key grazing families (Labridae, Acanthuridae, Siganidae, Pomacentridae, Balistidae, and Chaetodontidae) were counted with total counts and relative abundance calculated for each family, timepoint and site. When possible, the same observer completed all surveys to reduce observer bias in the data. Observer 1 completed > 55% of the surveys. The time, depth, tide, sea state, visibility and reef complexity were also recorded during surveys. Sea state and reef complexity were scored based on descriptions from the AIMS Long Term Monitoring Program^52^.

Feeding and grazing frequency within a 0.25 m^2^ plot around 2 haphazardly selected sets of seeding devices at each site were assessed using GoPro (HERO 9) video surveillance in the morning (8:30–11:30) and afternoon (14:00–17:00) of each survey timepoint (2, 90, and 240 d). GoPro cameras were mounted to lead dive weights using cable ties and left to record for 1–2 h in the absence of divers. The number of videos and hours of footage were standardized across sites and the fish grazing activity was recorded. The fish found to be feeding in the plots were identified to species and the following information was collected: number of feeding forays (attempts), number of bites per foray, device grazing (yes/no, per bite), plug grazing (yes/no, per bite), plot type (devices in pairs or triplicates depending on life-stage treatment), and device treatment (exclusion, featureless, or cage). Detailed information for video assessment criteria and data collection can be found in Supplementary Sect. 1.3 and Table S3.

### Benthic habitat and community assessments

Reef habitat assessments were undertaken at 4 spatial scales: plug, device, plot, and site level. Images of the coral seeding devices and plugs were taken at all timepoints to track the growth of benthic competitors across treatments and overtime. Within plots, 0.25 m^2^ quadrats were used to estimate the cover of benthic community constituents (crustose coralline algae, turf algae, fleshy macroalgae [to genus], cyanobacteria, live hard coral [to genus], soft coral, other benthic invertebrates, invertebrate grazers, and ‘bare’ substrate [defined as recently grazed algal turfs present on hard rock or rubble]). Quadrats were placed centrally around the devices and imaged (Olympus TG6) during all 3 timepoints (2, 90 and 240 d; Table S2). Images were imported into the Reef Cloud online database (https://reefcloud.ai/) and 25 randomly overlaid points per image were scored (human observer) to one of the benthic community categories. Image classifications (109–188 total images per site) were used for statistical analysis.

Site-level benthic data were obtained from in situ point-intercept surveys collected at the 2-d timepoint only (Table S2), from 3 replicate 30-m transects per site, with data recorded every 50 cm, allocated to the same benthic community constituent categories as above. During all 3 timepoints, a single 30-m video transect was recorded (Olympus TG6). Frame grabs (*n* = 35) were taken from video transects and scored (human observer) on Reef Cloud, with 10 randomly overlaid points per image. The in situ benthic data (at the site level) were compared to the image classifications (at the site level) and both data sets were investigated further using statistical analyses (see below).

### Environmental data: wave energy and sediment

Clod cards (i.e., small blocks made of plaster of Paris) were used to compare relative water flow among sites by measuring dissolution over time and comparing Diffusion Factors (*DF*^53^, defined as the ratio between the weight of material dissolved in an experimental block to a control block maintained in a stable environment)^53,54^. Clod cards were glued to the lid of a plastic food container and secured to a lead dive weight with cable ties for deployment. Five replicate clod cards were pre-weighed, then deployed to each site for 48–72 h (Table S2). Upon retrieval, the food containers were gently placed over the clods and secured to the lids before returning them to the surface. Clod cards were dried and the weight loss (defined as the card value [*CV*]^53^, pre- minus post-deployment weight) was used to obtain the dissolution rate (g m^−1^d^−2^). Weight loss was also used to calculate water velocity (cm s^−1^; *V* = [*CV*—5.8]/0.3)^53^. Values for *V* were then used to obtain *DF* (*DF* = 0.06V + 1.3)^53^. To reduce variability in the data due to sea state, clod cards were deployed on the same day at all sites.

Deposited sediments were assessed using two methods, ‘SedPods’ and ‘TurfPods’, that were created following techniques from Field et al. 2013^55^. Sediment collection pods (5 replicates per type) were deployed to sites (*n* = 4) at each survey timepoint (*n* = 3) and collected after 5–7 d (Table S2). From this, the total mass of deposited sediment was quantified for each pod type, which approximates sediment deposition on massive coral surfaces and algal turf-covered substrate, respectively. Pods were stored in a small volume of seawater at −20 °C and held until processing. Sediment processing followed standard water and wastewater methods from the American Public Health Association 2018 (see Supplementary Sec 1.4). Briefly, the individual samples were rinsed to eliminate salts (5–6 times with reverse osmosis water and a 3-h settlement period in between rinses), dried (24 h, 103–105 °C), and weighed to obtain the total mass per sample. Sediments were not separated by composition (organics or inorganics) or fractionated by particle size, due to the small mass obtained per sample.

## Statistical analyses

### Coral survival and predation from grazing fish

The coral survival and fish grazing data were analyzed using generalized linear mixed effect models in R statistical software^56^ using the ‘glmer’ function in the ‘lme4’ package^57^. To investigate the change in survival through time and among treatments and life-history stages, we first modelled survival (binomial distribution with a logit link function) against the additive effects of survey time point, coral life stage, and the interaction between device treatment and plug position, with a random effect of site. To investigate whether survival was different among sites, we then modelled survival (at the final timepoint only) against the additive effect of site, device type and life stage as fixed effects, with other variables (plug position, and device number) as random effects. Presence or absence of coral grazing after 2 d (binomial distribution with a logit link function) was modelled against coral life stage, site, and the interaction between device treatment and plug position as the fixed effects, with device number as the random effect. For each analysis, different combinations of predictors were tested, and the ‘best’ routine models were selected by comparing Akaike Information Criterion (AIC). Diagnostic plots of the residuals and Q-Q (DHARMa^58^) were used to validate model assumptions and test for homogeneity of variance and linearity between the predictors and the response variables. All parameters were compared and tested for significance using the ‘emmeans’ package^59^ and model outputs were plotted against the response for data visualization using the ‘ggplot2’ package^60^.

### Fish abundance and grazing behavior across seeding sites

Redundancy analyses (RDAs, R software^56^,’rda’ function of the ‘vegan’ package^61^) were used to explore the fish abundance and grazing data. From this, ordination plots were generated to identify the site or combinations of sites that explained the greatest differences in fish abundance and grazing intensity. Linear regression models (R software^56^, ‘lm’ function) were used to test the significance of fish families, genera, and species (abundance and grazing) against PC1 and PC2 of the RDAs. Additive combinations of species, genera and families were also tested. Likewise, site was regressed against PC1 and PC2 to test for its significance. Three zero-inflated generalized linear mixed effects models (R software^56^, ‘glmmTMB’ function and package^62^) were used to further explore fish abundance and grazing by site and fish taxonomic group. First, fish abundances and bites (as the response variables) were modeled against site and fish categories (family-level identification) as predictors. Then, bites by parrotfish (Labridae, Scarini) were tested alongside site and species-level categories. A truncated Poisson distribution was used to accommodate count data that might exhibit overdispersion while still constraining the values to be non-negative. A zero-inflated component was included to address the excess zeros commonly observed in count data.

### Ecological data: benthic composition, sedimentation, and wave energy

RDAs were conducted to investigate whether there was a clear separation in the benthic community composition among sites with higher and lower coral survival and grazing at the 2-d timepoint. The RDAs included abundance (percent cover of benthic community constituents per plot) against site, coral survival, or coral grazing as predictors, and visualized through ordination plots. A linear regression model was then used to investigate the relationship between PC1 and PC2, and the explanatory variables. Coral survival and grazing were also tested as the responses against PC1 and PC2 as additive predictors. Logistic regression models (glm) were used to test the abundance of significant benthic categories (groups or individual constituents) as predictors of coral survival and grazing. Models (lm and glm) were also built to investigate differences in wave energy and sedimentation among sites. Deposited sediments, diffusion rates, or diffusion factors were modeled against the predictors of site and pod type (for sediment data only). All models were run and validated as described above.

## Results

*Acropora digitifera* survival and grazing significantly differed by device treatment, orientation, life stage and site. Grazing pressure was also related to ecological attributes of the seeding site. Below we report the results of coral survival and grazing pressure, in turn, followed by the ecological variables that underpinned differences across sites. Please refer to Supplementary Tables S4 to S10 for the full results of statistical analyses.

### Coral survival

We identified four major results with regards to coral survival: (1) survival was highest on devices with cages followed by those with protective features, and lowest on featureless controls; (2) survival was higher on side- versus top-oriented plugs, (3) survival declined through time in all treatments; and (4) microfragments had higher survival than spat (Fig. 2).

Within 2 d of seeding, overall survival declined significantly (Fig. 2a). However, survival was twofold higher on exclusion devices than on featureless devices (GLMM, *p*-value = 0.002, estimate = 0.68, SE = 0.23, z-ratio = 2.98; Table S4; Fig. 2a). The most significant early decline was observed for coral spat on featureless devices (GLMM, *p*-value < 0.001, estimate = −1.13, SE = 0.21, z-ratio = −5.437; Table S4; Fig. 2a), where average survival dropped from 100 to 52%, and was as low as 0% at one site. Coral survival continued to decline over time in all treatments, with < 15% of spat in control devices surviving to 90 d (GLMM, *p*-value < 0.001, estimate = 2.15, SE = 0.25, z-ratio = 8.55; Table S4; Fig. 2a). After 240 days, there was up to 2-times higher survival in the exclusion treatment compared with featureless devices, and this result was strongest for coral spat (GLMM, *p*-value < 0.001, estimate = −1.13, SE = 0.21, z-ratio = −5.44; Table S4; Fig. 2a). Microfragments in caged devices had the highest survival at the end of the experiment (Fig. 2a).

Low survival of spat and high survival of microfragments was consistent across all sites and treatments (GLMM, *p*-value < 0.001, estimate = −3.15, SE = 0.3, z-ratio = −15.23; Table S4; Fig. 2a). Generally, microfragment survival remained high across the 240-d deployment. The highest survival achieved was in the caged treatment (97%) with lower survival in devices with features (85%) and significantly lower survival in control devices (60%; GLMM, *p*-value = 0.042, estimate = −2.94, SE = 0.73, z-ratio = −4.02; Table S4; Fig. 2a).

Average survival was significantly higher in side-facing corals than top-facing ones, and this pattern was consistent across device treatments (featureless and exclusion feature), life-history stages, and through time (GLMM, *p*-value = 0.001, estimate = −0.65, SE = 0.20, z-ratio = −3.30; Table S4; Fig. 2a). This result was most pronounced for microfragments, with 1.5-times more survivors on side versus top oriented corals after 240 d. However, the best performing orientation and device combination for microfragments was the upward-facing plug in the caged treatment (Fig. 2a). Survival in these positions was 1.6-fold higher than upward-facing plugs in featureless device (lowest performing orientation and device combination) but was not significantly different from side-facing plugs in the exclusion device.

After 240 d, there was no significant difference in spat or microfragment survival between sites (Fig. 2b). Site 1 had the highest number of surviving spat and microfragments at the end of the experiment, while Site 3 had the lowest (Fig. 2b); this result remained consistent across the device treatments.

### Effects of fish grazing

Within 2 d of seeding, a significant number of corals on featureless devices were heavily grazed by fishes and were grazed up to 4-times more often than those in the exclusion treatment (GLMM, *p*-value < 0.001, estimate = −2.72, SE = 0.57, z-ratio = −4.78; Table S5; Fig. 2c). Top-orientated corals were targeted for grazing significantly more often (up to 18-times more) than side orientations (GLMM, *p*-value = 0.035, estimate = −0.83, SE = 0.39, z-ratio = −2.11; Table S5; Fig. 2c), and significantly more spat were grazed than microfragments (GLMM, *p*-value = 0.03, estimate = 0.77, SE = 0.36, z-ratio = 2.1; Table S5; Fig. 2c)**.** Despite protection in the exclusion devices, up to 55% of spat and 25% of microfragments in the top orientation experienced grazing (Fig. 2c). However, the fish bites in exclusion devices were constrained to the outer edge of the top plug and therefore never caused 100% mortality. Overall, the device and orientation combination that experienced the least grazing was the side position of the exclusion device, while the most was the top position of the featureless device (GLMM, *p*-value = 0.01, estimate = −2.94, SE = 1.18, z-ratio = −2.5; Table S5; Fig. 2c).

Fish grazing was also site-specific. On average, corals had up to 5-times more grazing and predation attempts at Site 3 than Sites 1 and 2 (GLMM, *p*-value < 0.001, estimate = 3.48, SE = 0.71, z-ratio = 4.91; Table S5; Fig. 2c), regardless of device type, with up to 95% of spat and 75% of microfragments on featureless devices experiencing grazing there.

### Ecological characteristics of seeding sites

#### Fish abundance, diversity and feeding activity

**Figure 3:**
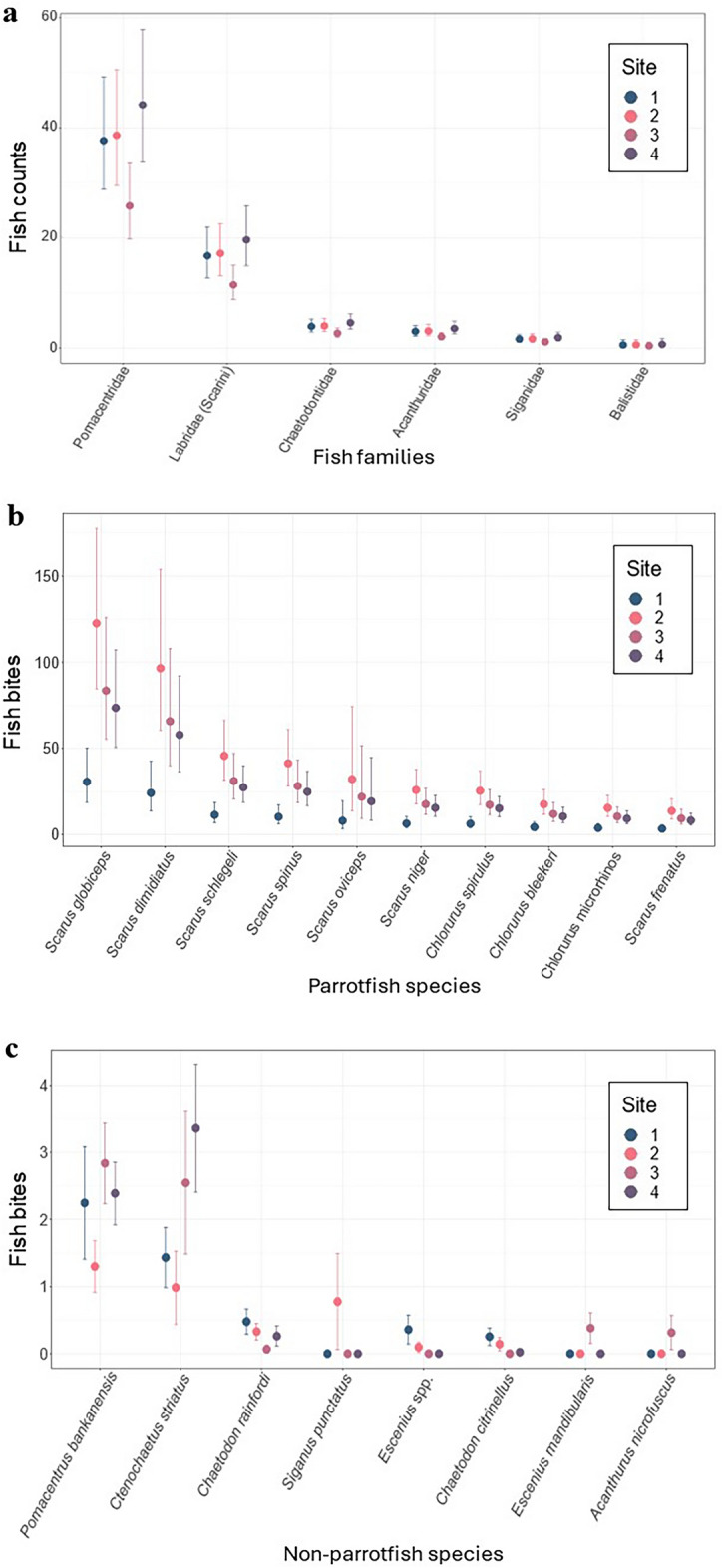
Site-level abundance and bite rates for common reef fishes. (**a**) Counts of fish from in situ stationary point count surveys and grouped by fish family (*n*_obs_ = 354, *n*_groups_ = 11; see Supplementary Table S6). (**b**) Feeding (bites min^-1^) by parrotfish, Labridae (Scarini), in 0.25 m^2^ plots within sites (*n*_obs_ = 8040, *n*_groups_ = 134; Table S7). (**c**) Fish bites (min^-1^) from non-parrotfish species in plots, separated by site. Standard error and 95% confidence intervals are displayed for each site for the observed (**c**) and modeled (**a**, **b**) data, respectively.

Differences in fish abundance, diversity and feeding activity among sites were evident. While some fish were consistently abundant across sites (i.e., Siganids, Balistids), others varied considerably (i.e., Pomacentrids, Labrids, Acanthurids and Chaetodontids). Site 3 had the lowest overall abundance of fish while Sites 1 and 4 had the highest abundance and diversity (Fig. 3a). For example, Sites 1 and 4 had nearly double the number of Chaetodontids and Acanthurids than Site 3 (GLMM, Site 1 to 3: *p*-value < 0.001, estimate = 0.4, SE = 0.05, z-ratio = 7.44, Site 3 to 4: *p*-value < 0.001, estimate = −0.54, SE = 0.05, z-ratio = −10.48; Table S6; Fig. 3a). Parrotfish abundance was also significantly higher at Sites 2 and 4 than at Site 3 (GLMM, Site 2 to 3: *p*-value < 0.001, estimate = 0.4, SE = 0.05, z-ratio = 7.94, Site 3 to 4: *p*-value < 0.001, estimate = −0.5, SE = 0.05, z-ratio = −10.49; Table S6; Fig. 3a).

Bites by scraping and excavating parrotfish (genera *Scarus* and *Chlorurus*) accounted for 55–85% of the total bites observed in device plots (Table S8). Whilst grazing directly on coral plugs was highest at Site 3 and lowest at Site 2 (Fig. 2c), the grazing activity in experimental plots, recorded during video surveillance, was highest at Site 2 (Table S8). A total of 9233 bites (8480 from parrotfish) were recorded at Site 2. That is approximately 47 bites min^−1^ m^−2^, and 2800 bites hr^−1^ m^−2^, up to 5.6-fold higher than other sites. Site 1 had significantly fewer bites (< 600 bites hr^−1^ m^−2^) than other sites, and this was strongest when comparing bites by parrotfish (GLMM, Site 2 to 1: *p*-value < 0.001, estimate = 1.38, SE = 0.31, z-ratio = 4.43, Site 3 to 1: *p*-value = 0.002, estimate = −0.5, SE = 0.33, z-ratio = 3.07; Site 4 to 1: *p*-value = 0.005, estimate = 0.87, SE = 0.31, z-ratio = 2.78; Table S7).

*Scarus globiceps* had the highest number of bites by parrotfish in device plots, while *S. dimidiatus*, *S. schlegeli*, *S. spinus*, *S. oviceps*, *S. niger*, *S. frenatus*, *Chlorurus spirulus*, *C. bleekeri*, and *C. microrhinos* accounted for the rest (Fig. 3b). When regressed against the principal components of total bites, *Scarus globiceps*, *S. schlegeli*, and *Chlorurus spirulus* made a significant contribution to variation in bites observed among sites (lm, *S. globiceps*: *p*-value < 0.001, estimate = 0.002, SE = 0, statistic = 13.94, *S. schlegeli*: *p*-value < 0.001, estimate = 0.01, SE = 0, statistic = 19.42, *C. spirulus*: *p*-value < 0.001, estimate = 0.01, SE = 0.001, statistic = 9.25; Table S7). All Scarini species were found to feed more often at Site 2 and by contrast, less often at Site 1 (Fig. 3b). The species *S. globiceps*, *S. dimidiatus* and *S. schlegeli* were identified as the top feeders (Fig. 3b).

Feeding contributions from other parrotfish (*S. altipinnis*, *S. chameleon, S. rubroviolaceus*, and *S. flavipectoralis)* and non-parrotfish species (*Pomacentrus bankanensis*, *Ctenochaetous striatus*, *Chaetodon rainfordi*, *Siganus punctatus* and two *Escenius* species) were low (6–200 bites hr^−1^ m^−2^; Table S8; Fig. 3c). The parrotfish fed more often at Site 2 while feeding results from other species were variable among sites.

#### Benthic composition, reef hydrodynamics, and sedimentation

**Figure 4:**
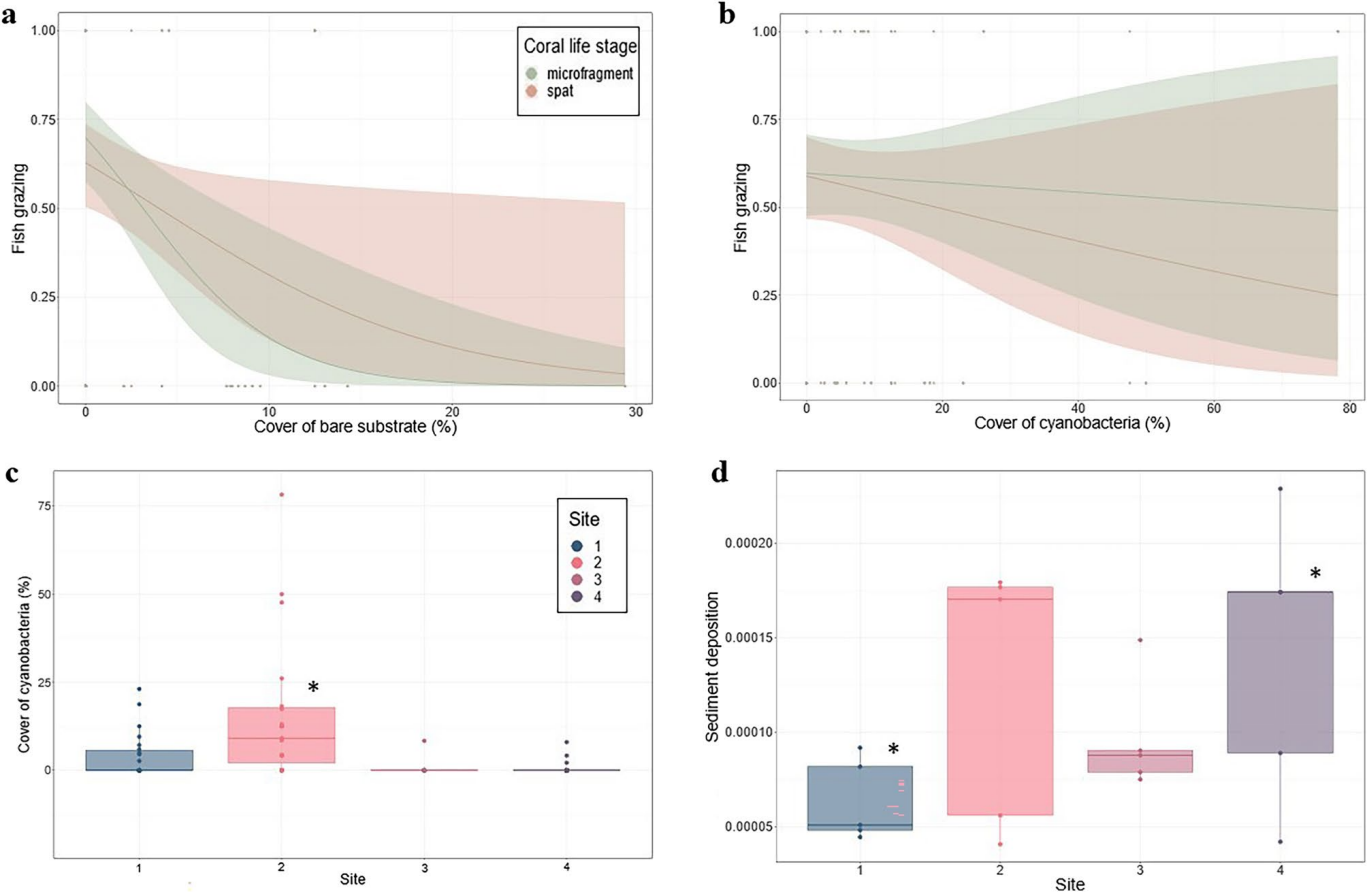
Ecological data collected across sites, compared to coral grazing and survival. Logistic relationships between the cover of bare substrate (**a**) or cyanobacteria (**b**) and the grazing of coral spat and microfragments by fishes at the 2-d timepoint. (**c**) Percent cover of mat-forming cyanobacteria recorded in device plots (0.25 m^2^, *n* = 31–41) in sites (*n* = 4) at the 2-d timepoint. (d) Sediment deposition (mg m^-2^ d^-1^) collected from Sed Pods (*n* = 15) deployed to sites (*n* = 4).

The composition of benthic organisms in experimental plots was related to fish grazing and coral survival. Fish grazing (on featureless devices at 2 d) was negatively related to the percentage cover of bare substrate; this was true across all sites and coral life stages (Fig. 4a). Increasing cover of bare substrate to 20% (0.25 m^2^) reduced grazing of seeded corals to < 10% (Fig. 4a). The cover of hard rock rather than rubble contributed most to this. Site 1 (with low grazing-induced mortality and low parrotfish feeding; Figs. 2c and 3b, respectively) had significantly more hard rock compared to other sites (lm, Site 2 to 1: *p*-value = 0.004, estimate = −4.18, SE = 1.40, t-value = -2.98, Site 3 to 1: *p*-value = 0.006, estimate = −4.01, SE = 1.42, t-value = −2.82, and Site 4 to 1: *p*-value < 0.001, estimate = −4.97, SE = 1.40, t-value = −3.54; Table S9 and Fig. S2).

Site 2, with low grazing-induced mortality (Fig. 2c) and high parrotfish feeding (Fig. 3b), was the only site with a significantly different benthic community (lm, p-value < 0.01, estimate = 0.659 SE = 0.233; Table S9). Most extreme was the difference in cyanobacteria, which was > 12 times higher at Site 2 than other sites (lm, Site 2 to 1: *p*-value < 0.001, estimate = 12.05, SE = 3.46, t-value = 3.48, Site 2 to 3: *p*-value < 0.001, estimate = 15.64, SE = 3.63, t-value = 4.31, Site 2 to 4: *p*-value < 0.001, estimate = 15.35, SE = 3.58, t-value = 4.28; Fig. 4c). Fish grazing on spat was negatively related to the percent cover of cyanobacteria (Fig. 4b). The most extreme difference in cyanobacteria cover was observed between Sites 2 and 3; high cyanobacteria cover appeared to cause less grazing and higher survival of spat at Site 2 than Site 3 (lm, spat grazing Site 3 to 2: *p*-value < 0.001, estimate = 0.81, SE = 0.17, t-value = 4.88, and spat survival Site 3 to 2: *p*-value = 0.009, estimate = −0.54, SE = 0.17, t-value = −3.21; Table S9).

Spat grazing and survival was also related to the abundance of *Acropora* corals with corymbose and digitate morphologies. For example, a 10% increase in *Acropora* corals was associated with a 10% decrease in the likelihood of grazing on spat and increased the chance of survival at Site 1 (Fig. S1). Site 1 had the highest overall cover of *Acropora* corals in plots, while Site 3 had the lowest. The influence *Acropora* coral cover on spat survival was most extreme when comparing Site 3 to Site 2 and 1, respectively (lm, Site 3 to 2: *p*-value = 0.01, estimate = −0.55, SE = 0.18, t-value = −3.03, and Site 3 to 1: *p*-value = 0.03, estimate = −0.28, SE = 0.11, t-value = -2.65; Table S9). The abundance of non-*Acropora* corals, octocorals, algal turfs and CCA contributed to site-related differences; however, not all variables correlated with coral grazing and/or survival, and the responses weren’t consistent among sites (Table S9).

There was little to no change in the composition of benthic communities on plugs throughout the experiment (including caged devices). The dominant benthic category at the time of deployment (i.e., those that developed in ex-situ aquaria) remained dominant at the end of the experiment.

Environmental data revealed slight variation in water movement and sediment deposition at the site level. Overall, the range of water motion was low across sites at the 2-d timepoint (*DF* of 3.68 to 5.42 on a *DF* scale of 1 [low] to 20 [high]; Table S10; Fig. S3). The total mass of deposited sediments on Sed Pods explained more of the among-site variation than sediment accumulation on the Turf Pods. On average, the deposited sediments collected from Sed Pods at Site 1 were lower than sediments at other sites (Fig. 4d) and Site 4 had significantly more sediments (lm, Site 4 to 1: *p*-value = 0.037, estimate = 7.8e^−05^, SE = 3.5e^−05^, statistic = 2.27; Fig. 4d; Tabe S10).

## Discussion

The experimental results indicated four ways to maximize survival of *Acropora digitifera* corals over an eight-month seeding trial: (1) deploy devices with fish-exclusion features; (2) orient corals in the side rather than top positions; (3) use differential survival data to inform appropriate microfragment and spat seeding densities to achieve target numbers; and (4) use ecological variables, like fish abundance, fish diversity and the abundance of nutrient-dense food sources for fish, to help select seeding sites. These four findings can be used to guide coral seeding methods and manage restoration outcomes.

Corals deployed on seeding devices without protection were the most vulnerable to grazing and suffered significantly higher mortality compared to those with protection. Complex device topographies, including crevices, microrefugia and those with high rugosity, have previously been shown to improve coral larval settlement and post-settlement survival^11,23,24,30^. Thus, it was not unexpected that the seeding device engineered with protective walls also enhanced coral survival after seeding. Although the caged device still resulted in higher survival of coral microfragments in our study the built-in protection provided by a fish-exclusion device offers a simple and effective alternative to reduce grazing pressure that is cheaper and far more scalable than cages. Furthermore, cages are ineffective against fish grazing after removal^33^. Nevertheless, there is room to improve the design of devices with features to offer more protection and further increase benefits over caged devices.

The orientation of corals in devices had a major influence on post-seeding survival. Coral larvae often preferentially settle on vertical surfaces to reduce the likelihood of disturbance-induced mortality during recruitment^63–66^. Our findings confirmed that corals (especially spat) deployed on top-facing plugs suffer higher rates of grazing and mortality than those in side-facing positions. Many species of parrotfish prefer to feed on horizontal and convex surfaces over vertical and concave surfaces^67^. Therefore, the vertical orientation of the side-facing plug may offer optimal refuge from some grazers. Our grazing results support the selection of vertical habitats by corals. However, there are also reasons beyond fish predation as to why coral larvae prefer settling in refugia. Coral spat are sensitive to high light, heavy sedimentation and excessive waterflow^68^, and vertically-orientated structures provide a buffer against such conditions. Fast growing turf, crustose, and macroalgae, that tend to out-compete corals in high-light and sediment-laden environments, can also be less abundant and display different morphologies (e.g., thin- versus thick-crusted CCA) in low-light environments^39,69^, relieving some competitive pressures. Regardless of the mechanisms, offering vertically oriented refugia for corals was a successful strategy for improving survival.

It was clear that coral life stage impacted survival after seeding, with significantly more *A. digitifera* microfragments surviving than spat. Spat survival was low (< 50%) on featureless devices past two days, and even with device protection. Low survival of spat is common in coral seeding experiments to date. For example, in French Polynesia, grazing led to mass mortality of *A. striata* spat after one-week of seeding^70^. Corals in cages had the highest survival but less than 40% survived beyond the initial week of deployment^70^. Similarly, in the Philippines, survival of *A. tenuis* spat was < 25% three-months post seeding and spat in partial cages outperformed those in full cages in this experiment^28^. By contrast, on the GBR, 38–65% of *A. cytherea* spat survived on reefs for one month, with caging increasing survivorship by 22%^27^. Finally, partial protection of *A. tenuis* spat in microrefugia led to 22–39% yield (e.g., device-level survival) after one year on the GBR^11,12^. Although these examples demonstrate that protection from fishes can increase survival during early ontogeny, spat survival typically remains well below 50%. We suggest that: (i) more spat per device need to be deployed to meet required survival outcomes, (ii) other device types should be trialed to improve the design of protective features, and (iii) further ecological drivers should be studied to inform optimal conditions for spat success after seeding.

While microfragmentation has almost exclusively been performed on corals with plating, massive and sub-massive growth morphologies^18,19^, our results indicate that the technique is also feasible when applied to branching taxa such as *Acropora*. Survival of *A. digitifera* microfragments was exceptionally high, greater than 75% on average and up to 97% in cages. These findings, alongside the result of previous experiments^30^, confirm the benefits of physical barriers to enhance the survival of coral microfragments. In Palau, small fragments of *Porites lobata* had higher survival when fully protected (> 90%, four-sided crevices on tiles) compared to the result of partial crevices (70%) and exposed controls (28%) after 29 days^30^. In the same study, *Pocillopora damicornis* fragments also initially benefited from protection; however, all had died after eight days. These outcomes indicate that tolerance to microfragmentation may be species specific and this will have flow-on effects to survival. Similarly, other factors including fragment health might dictate the likelihood of predation post deployment, with recently dead or dying tissues being more attractive to fishes^71^. The *A. digitifera* tested in our study may be more tolerant to microfragmentation and less palatable to fishes, making it a target candidate for seeding over other taxa such as *Pocillopora*. To ensure positive outcomes after seeding, adequate time should be provided for corals to heal after the microfragmentation process, and the palatability of a range of coral species should be examined.

We found that coral spat were also targeted for grazing more often than microfragments. Parrotfish, mainly *Scarus globiceps*, were identified as the most common and destructive grazers of seeded corals, especially spat. However, most scraping parrotfish are nominally herbivorous and therefore we hypothesize that the grazing-induced mortality of coral spat in our study is likely accidental and indirect. The morphological description of the jaw of a scraping parrotfish like *S. globiceps*, confirms their ability to graze early successional bacterial-algal biofilms growing on reef substrata^67,72^. The artificial plugs used to grow corals in our study were conditioned with similar biofilms (e.g., thin-crusted CCA, microalgae and mixed bacteria) and these are known to be palatable to many herbivorous parrotfishes^73,74^. Thus, it is likely parrotfish were targeting the benthic community growing on the plug rather than the coral itself. Furthermore, spat plugs were held in aquaria longer than microfragment plugs prior to deployment; this likely contributed to significant differences in grazing among the coral life stage treatment. The use of less palatable benthic organisms for plug conditioning and the incorporation of an antifoulant (i.e., chemical deterrent) on plugs or devices are potential direct and indirect solutions to deter parrotfish grazing. However, we suggest these methods must be balanced against the requirement for benthic communities that: (i) are inductive for the larvae during settlement, (ii) reduce competition against spat and microfragments during recruitment, and (iii) promote coral survival to maturity.

Lastly, the influence of grazing on survival is strongly site-specific, and this information may be useful to guide the selection of sites for successful seeding. Foremost, our results support growing evidence that herbivorous parrotfish may preferentially target nutrient-rich food sources (e.g., algal mats or turfs dominated by cyanobacteria), when available^75,76^. However, on reefs with high coral cover and low abundance of cyanobacteria, opportunistic grazing on less preferred food sources such as deployed corals may occur. For example, Site 2 exhibited the least grazing on coral plugs but the highest number of parrotfish bites in experimental plots, and concurrently, a notably higher (12 times) abundance of mat-forming cyanobacteria. Parrotfish consumption of nutrient-dense foods, such as endolithic algae and cyanobacteria, has been documented in the Caribbean^75^ and the northern GBR^76^. Field observations performed by Cissell et al.(2019) found that greater than 15% of the diet of parrotfishes *S. iseri* and *S. coeruleus* consisted of mat-forming cyanobacteria^75^. Similarly, stable isotope analysis of 22 species of GBR-dwelling parrotfish provided strong evidence to support the consumption and preference of microscopic cyanobacteria over other algal substrates during feeding^76^. From this, we propose a new hypothesis that deployment adjacent to nutritious foods like cyanobacteria can offer natural forms of refuge to corals from grazing after seeding.

*En masse*, deploying to sites with high-nutrient food sources for fish may limit both direct and indirect grazing pressure on corals, increasing the likelihood of successful restoration. However, the composition of modern reefs is expected to shift as the frequency of mild to severe disturbances increases over time. For example, sudden spikes in the abundance of mat-forming cyanobacteria are common characteristics of recently disturbed reefs^77,78^. We suspect slight increases in cyanobacteria (< 20% in 0.25 m^2^) can aid coral survival after seeding; however, too much cyanobacteria can negatively impact coral dominance on disturbed reefs. For *A. digitifera* spat, we found that a minimum cover of closely related Scleractinian corals (10%) and bare rock (20%) was correlated with a reduction in grazing by 10 to 50%, respectively. Indeed, other benthic constituents, like sponges and epilithic algal communities, have been correlated with high survival of seeded corals on the GBR^12^. Therefore, a diverse group of grazing fishes at a minimum biomass may be required to mediate competition between corals and fast-growing benthic organisms after seeding. A relatively low biomass of herbivores (177 kg ha [17.7 g m^2^]) has been shown to sustain community equilibrium and reduce the risk of regime shifts occurring in the Indian Ocean^79^. Defining a biomass threshold for parrotfish (e.g., where the net benefit towards coral competition outweighs the negative effects of accidental grazing), represents one tool that can be used to guide or tailor deployments of corals to specific sites. The direct manipulation of herbivore abundance (i.e., enhancing fish recruitment) could also be considered at sites with significant regime shifts. Taken together, these results highlight the importance of a holistic approach that considers multiple ecological parameters to inform the selection of candidate sites for reef restoration with coral seeding devices.

## Conclusion

The addition of fish-exclusion features in coral seeding devices can improve survival and lead to substantial reductions in the handling time required for coral seeding, supporting the goal of upscaling restoration initiatives. Our eight-month seeding trial with *Acropora digitifera* revealed significantly higher survival when spat and microfragments were deployed in vertical positions with protection from grazing fishes. Importantly, future coral seeding experiments need to consider biological and environmental characteristics of the receiving environment more holistically and test across ecologically important reef-building coral taxa. Here we’ve identified that environmental drivers including a diverse assemblage of fishes (Labrids, Pomacentrids, Acanthurids, Chaetodontids) and low to moderate cover (< 40%) of coral, recently grazed reef rock, and nutrient-rich foods for parrotfish, could be used as visual aids (i.e., during pre-deployment assessments) to optimize the selection of sites for seeding in the future. While the role that herbivorous fishes play in driving coral-seeding success requires further investigation, our results suggest that restoration research can be used both to guide the development of coral seeding techniques and to advance our fundamental understand of reef ecology.

### Supplementary Information

Supplementary Information.

## Data Availability

All data is available through the Australian Institute of Marine Science public-data repository at https://apps.aims.gov.au/metadata/view/7e4d3f91-a85f-47e1-802c-09b506f21fe5.
