# Supplementary material for: Coral-seeding devices with fish-exclusion features reduce mortality on the Great Barrier Reef
Source: Sci Rep. 2024 Jun 10;14:13332. doi: 10.1038/s41598-024-64294-z (PMC11165004; doi:10.1038/s41598-024-64294-z)
Supplement: Supplementary file 1 — Supplementary Information. [file 41598_2024_64294_MOESM1_ESM.docx]

Coral-seeding devices with fish-exclusion features reduce mortality on the Great Barrier Reef

**Authors:** *TN Whitman, MO Hoogenboom, AP Negri, CJ Randall

**Supplementary materials**

**Methods**

**1.1. Spawning, larval culture and settlement**

**Table S1.** Spawning, larval culture and settlement information for 13 *Acropora digitifera* broodstock colonies collected for autumn coral spawning in March 2021. An asterisk * identifies the larval culture used for seeding due to high settlement success and strong post-settlement survival.

| ***Spawning and Culture Information*** | | | | | |
| --- | --- | --- | --- | --- | --- |
| **Colony** | **Spawn Date**  **(d/m/yyyy)** | **Time** | **Spawn Amount** | **Culture Number** | **Gamete Contribution** |
| **1** | 9/3/2021 | 21:27-22:30 | Partial | 0* | Egg and Sperm |
|  | 10/3/2021 | 21:42-22:37 | Partial | 3 | Egg and Sperm |
| **2** | 10/3/2021 | 21:49-22:37 | Full | 1 (A and B) | Egg |
|  |  |  |  | 2 (A and B) | Sperm |
| **3** | 9/3/2021 | 21:27-22:30 | Partial | 0* | Egg and Sperm |
|  | 10/3/2021 | 21:38-22:37 | Full | 1 (A and B) | Egg |
|  |  |  |  | 2 (A and B) | Sperm |
|  |  |  |  | 3 | Egg and Sperm |
| **4** | 10/3/2021 | 21:43-22:37 | Full | 1 (A and B) | Egg |
|  |  |  |  | 2 (A and B) | Sperm |
|  |  |  |  | 3 | Egg and Sperm |
| **5** | 10/3/2021 | 21:45-22:37 | Full | 1 (A and B) | Egg |
|  |  |  |  | 2 (A and B) | Sperm |
|  |  |  |  | 3 | Egg and Sperm |
| **6** | 10/3/2021 | 21:40-22:37 | Partial | 0* | Egg and Sperm |
|  |  |  |  | 2 (A and B) | Egg |
|  |  |  |  | 3 | Egg and Sperm |
| **7** | 10/3/2021 | 21:55-22:37 | Full | 1 (A and B) | Sperm |
|  |  |  |  | 2 (A and B) | Egg |
|  |  |  |  | 3 | Egg and Sperm |
| **8** | 10/3/2021 | 21:38-22:37 | Full | 1 (A and B) | Sperm |
|  |  |  |  | 2 (A and B) | Egg |
|  |  |  |  | 3 | Egg and Sperm |
| **9** | 9/3/2021 | 21:27-22:30 | Nearly full | 0* | Egg and Sperm |
|  | 10/3/2021 | 21:45-22:37 | Full | 1 (A and B) | Egg |
|  |  |  |  | 2 (A and B) | Sperm |
|  |  |  |  | 3 | Egg and Sperm |
| **10** | 10/3/2021 | 21:49-22:37 | Full | 1 (A and B) | Sperm |
|  |  |  |  | 2 (A and B) | Egg |
|  |  |  |  | 3 | Egg and Sperm |
| **11** | 10/3/2021 | 21:50-22:37 | Partial | 2 (A and B) | Egg |
|  |  |  |  | 3 | Egg and Sperm |
| **12** | 10/3/2021 | 21:45-22:37 | Partial | 1 (A and B) | Sperm |
|  |  |  |  | 2 (A and B) | Egg |
|  |  |  |  | 3 | Egg and Sperm |
| **13** | 10/3/2021 | 21:45-22:37 | Full | 1 (A and B) | Sperm |
|  |  |  |  | 2 (A and B) | Egg |
|  |  |  |  | 3 | Egg and Sperm |

Field collected CCA specimens (target genera: *Porolithon*, *Lithophyllum*, and *Titanoderma*) were used to accelerate the plug-conditioning process. The CCA specimens were collected from Davies Reef (February 2021) in compliance with permit G21/38062.1 issued by the Great Barrier Reef Marine Park Authority. After 2 months, the frag plugs comprised an early-successional and mixed community of CCA and biofilms. The CCA and bacteria were not classified to species but were sufficiently inductive, with high numbers of larvae settled on plugs after 24 h (~9 spat per plug).

**1.2. Biological and environmental data collection**

**Table S2.** Site location and data collection for the coral seeding experiment at Davies Reef. Data collection was attempted over three timepoints 2, 90 and 240 days after seeding and at all four sites. The “**✓**” reflects a successful collection of data at all sites, while the “⌧” refers to an unsuccessful attempt at all four sites. Poor weather, due to winds, tides, and waves was the main cause of unsuccessful data collection.

| **Site Name**  *(Davies Reef, mid shelf, Great Barrier Reef)* | | **Site Coordinates** | |
| --- | --- | --- | --- |
|  |  | **Latitude** | **Longitude** |
| Site 1 | | -18.826097° | 147.627017° |
| Site 2 | | -18.818100° | 147.632533° |
| Site 3 | | -18.819533° | 147.631233° |
| Site 4 | | -18.820600° | 147.630633° |
| **Data Collection** | **Days Seeded** | | |
|  | **2 days** | **90 days** | **240 days** |
| Coral survival (plug images) | **✓** | **✓** | **✓** |
| Fish grazing (plug images) | **✓** | ⌧ | ⌧ |
| Fish counts (diver survey) | **✓** | **✓** | **✓** |
| Fish feeding (video survey) | **✓** | **✓** | **✓** |
| Benthic quadrat (images) | **✓** | **✓** | **✓** |
| Benthic transect (images or video) | **✓** | **✓** | **✓** |
| Benthic transect (diver survey) | **✓** | ⌧ | ⌧ |
| Sediment pods (deployment and retrieval) | **✓** | **✓** | ⌧ |
| Clod cards (deployment and retrieval) | **✓** | **✓** | ⌧ |

**1.3. Fish video selection process and data recording for grazing pressure**

**Table S3.** Detailed information from the GoPro video analysis to quantify grazing pressure. Categories are explained via definitions and possible response. Data entries were taken for each individual fish that was observed to be grazing in the experimental plot. Not all categories were used in the data analysis.

| ***GoPro Video Analysis: Data collection metrics, descriptions, and potential responses*** | | |
| --- | --- | --- |
| **Metric** | **Description** | **Possible Response** |
| Sea state | Refers to the state of the water with regards to the weather conditions due to wind, waves, and current. | Calm (0-1.1 km/h), Light (1.1-12.6 km/h), Gentle (12.6-19.8 km/h), Moderate (19.8-28.8 km/h), Fresh (28.8-38.9 km/h) |
| Visibility | Referring to the sea state, regarding the distance (m) a diver/data recorder can visibly see and identify objects. | < 5m, 6-10m, 11-15m, 16-20m |
| Family | Refers to the taxonomic family for each fish identified. | Labridae, Pomacentridae, Acanthuridae, Blastidae, Blennidae, Chaetodontidae, etc. |
| Interval | A number that refers to the feeding approach and start of a feeding foray for an identified fish. The interval stops when a fish swims up, away from the substrate, and can have 1 or more bites. | Integer value, ranging from 1 to 50 |
| Bites | The number of bites a fish takes during a single feeding foray/interval. | Integer value, ranging from 1 to 75 |
| Grazing location | The location of the identified fish bite/graze. | Substrate, device, or plug |
| Plot type | Refers to the number of devices in each video and experimental plot, either three devices (cage, control, and exclusion) or two devices (control and exclusion). | Triplicate or pair |
| Time | Refers to the time the fish swims on screen and into the video frame. Screenshot images were taken of each individual fish for identification at this time point. | Between 1-12 minutes (e.g. 01:30) |
| Comments | Anything worth noting that was observed in the video. | Aggressive damselfish, invertebrate grazer in plot, diver present on video, camera knocked from original placement point, transect tape or star picket in view, etc. |

Replicate video clips (n=6-12; 12 min recording per clip) per replicate GoPro (n=2) per deployment (n=2, 1 morning and 1 afternoon) were captured for each site for each of three timepoints (2, 90 and 240 days) after coral seeding. In some cases, the videos files became corrupted or damaged. This resulted in a total of 33-35 replicate clips (396-420 minutes of footage) per site used from two time points (2 and 240 days) in the final analysis, and included a minimum of six replicate video clips, one replicate GoPro and one deployment per timepoint.

Video clips were analyzed individually using VLC media player software. The first and last clip in a GoPro deployment series were avoided to ensure fish remained undisturbed by divers (i.e., only clips 2-11 were used if 12 clips were recorded on a camera). A fish of interest was defined as an individual grazing within “two device lengths” from the seeding devices in the video frame, which encompassed the 0.25 m^2^ experimental plot where devices were fixed to the reef. For each grazing fish seen, the following data were collected: (1) species, (2) number of bites per feeding foray (i.e., total bites per feeding attempt), (3) the location of the bite (substrate, device or plug), and (4) the plot type (triplicate or pair). Therefore, if a fish swam out of the frame and came back, it was counted as a new individual. One observer was used to reduce variability in the data. Fish images (screen grabs from video clips) and video times were used by a second observer for quality control checks and fish species identification. The data were expressed per video clip as bites per species and bites per minute. The environmental conditions were also recorded for each clip to better explain outliers in the data set (Table S3).

**1.4. Sediment sampling and processing**

Sediments deposition on deployed pods (Sed Pod and Turf Pods) were rinsed into individual clean containers (2 L plastic Tupperware). A minimum of five rinses with Reverse Osmosis (RO; 1 L) water were used to remove salts from samples. In between rinses a minimum of 3 hours settling time was used to ensure all sediment material had fallen out of suspension. Once sediments had settled out the RO water was siphoned off (Air Admiral® P-79202-05, 230V, 50Hz) and 1 L of new RO water was added. The final rinse included a 24-hr settlement period. After five rinses, the samples were placed in the oven (Microprocessor Temperature Controller, Clayson OM1000) at 103-105 °C for 24 hrs. The dried samples were then placed in a glass vacuum desiccator (United Scientific DSGL150) with silica beads for 1 hr and then weighed (analytical balance, KERN Pes version 1.6) to calculate a total mass. Prior to sample drying, aluminium dishes were pre-heated in the oven (103-105 °C for 1hr) then cooled in the desiccator for 1hr. Weight of the aluminium dishes were taken, and this was used as the pre-sample weight. Total mass was calculated by subtracting the pre-sample weight (aluminium dish only) from the end sample weight (aluminium dish with sediment sample). Prior to sampling, visual inspections of pod, pod cap and collection bags (2 L plastic freezer bags) were conducted to identify tears, leaks and/or damage to the pod, and these samples were discarded.

**Results**

**2.1. Coral survival**

**Table S4.** Model output for coral survival data. Generalized linear mixed effects (GLMM; R Software) models were used with coral survival as the response and device type, plug position, survey timepoint or site as predictors; the exact model used for each output is defined in the table. Significance codes represent p values with “*” as <0.05, “**” as <0.01, and “***” as <0.001.

| **Coral Survival Models** | | | | | |
| --- | --- | --- | --- | --- | --- |
| **Model 1** | | | | | |
| **GLMM fit by maximum likelihood (Laplace Approximation)** | | | | | |
| Family: binomial (logit) |  |  |  |  |  |
| Formula: Survival ~ DeviceType * PlugPosition + Timepoint + LifeStage + (1 \| Site)  No. Observations: 2456  No. Groups: Site, 4 | | | |  |  |
|  |  |  |  |  |  |
|  | **estimate** | **std.err** | **z.value** | **p.value** | **signif.code** |
| (Intercept) | 21.585 | 24.778 | 0.871 | 0.384 |  |
| Device Type Exclusion | 0.678 | 0.228 | 2.979 | 0.003 | ** |
| Device Type Cage | 1.351 | 0.489 | 2.765 | 0.006 | ** |
| Plug Position Top | -0.653 | 0.198 | -3.298 | 0.001 | *** |
| Timepoint 2 | -17.676 | 24.778 | -0.713 | 0.476 |  |
| Timepoint 90 | -19.831 | 24.778 | -0.800 | 0.424 |  |
| Timepoint 240 | -20.472 | 24.778 | -0.826 | 0.409 |  |
| Life Stage spat | -3.154 | 0.207 | -15.232 | 0.000 | *** |
| Device Type Exclusion: Plug Position Top | 0.452 | 0.304 | 1.485 | 0.138 |  |
| Device Type Cage: Plug Position Top | 1.584 | 0.876 | 1.809 | 0.070 |  |
| **Model 1: *Planned comparisons*** | | | | | |
| **Device Type** |  |  |  |  |  |
| *Results for coral spat* | **estimate** | **std.err** | **z.value** | **p.value** | **signif.code** |
| Exclusion Side - Control Top (Timepoint 2) | 1.331 | 0.217 | 6.145 | 0.000 | *** |
| Control Top - Exclusion Top (Timepoint 2) | -1.130 | 0.208 | -5.437 | 0.000 | *** |
| Control Top - Exclusion Top (Timepoint 90) | -1.130 | 0.208 | -5.437 | 0.000 | *** |
| Exclusion Side - Control Top (Timepoint 240) | 1.331 | 0.217 | 6.145 | 0.000 | *** |
| Control Top - Exclusion Top (Timepoint 240) | -1.130 | 0.208 | -5.437 | 0.000 | *** |
| *Results for coral microfragments* | **estimate** | **std.err** | **z.value** | **p.value** | **signif.code** |
| Control Top - Exclusion Top (Timepoint 2) | -1.130 | 0.208 | -5.437 | 0.000 | *** |
| Control Top - Cage Top (Timepoint 2) | -2.935 | 0.730 | -4.021 | 0.042 | * |
| Control Top - Exclusion Top (Timepoint 90) | -1.130 | 0.208 | -5.437 | 0.000 | *** |
| Control Top - Cage Top (Timepoint 90) | -2.935 | 0.730 | -4.021 | 0.042 | * |
| Exclusion Side - Control Top (Timepoint 240) | 1.331 | 0.217 | 6.145 | 0.000 | *** |
| Control Top - Exclusion Top (Timepoint 240) | -1.130 | 0.208 | -5.437 | 0.000 | *** |
| Control Top - Cage Top (Timepoint 240) | -2.935 | 0.730 | -4.021 | 0.042 | * |
| **Time** |  |  |  |  |  |
| *Results for coral spat* | **estimate** | **std.err** | **z.value** | **p.value** | **signif.code** |
| *2 vs. 90 days* |  |  |  |  |  |
| Control Side - Control Side \| Control Top - Control Top | 2.155 | 0.252 | 8.550 | 0.000 | *** |
| Exclusion Side - Exclusion Side \| Exclusion Top - Exclusion Top | 2.155 | 0.252 | 8.550 | 0.000 | *** |
| *90 vs. 240 days* |  |  |  |  |  |
| Control Side - Control Side \| Control Top - Control Top | 0.641 | 0.160 | 4.019 | 0.042 | * |
| Exclusion Side - Exclusion Side \| Exclusion Top - Exclusion Top | 0.641 | 0.160 | 4.019 | 0.042 | * |
| *Results for coral microfragments* | **estimate** | **std.err** | **z.value** | **p.value** | **signif.code** |
| *2 vs. 90 days* |  |  |  |  |  |
| Control Side - Control Side \| Control Top - Control Top | 2.155 | 0.252 | 8.550 | 0.000 | *** |
| Exclusion Side - Exclusion Side \| Exclusion Top - Exclusion Top | 2.155 | 0.252 | 8.550 | 0.000 | *** |
| Cage Top - Cage Top | 2.155 | 0.252 | 8.550 | 0.000 | *** |
| *90 vs. 240 days* |  |  |  |  |  |
| Control Side - Control Side \| Control Top - Control Top | 0.641 | 0.160 | 4.019 | 0.042 | *** |
| Exclusion Side - Exclusion Side \| Exclusion Top - Exclusion Top | 0.641 | 0.160 | 4.019 | 0.042 | *** |
| Cage Side – Cage Side \| Cage Top - Cage Top | 0.641 | 0.160 | 4.019 | 0.042 | *** |
| **Microfragment vs. Spat** | **estimate** | **std.err** | **z.value** | **p.value** | **signif.code** |
| *2 days* |  |  |  |  |  |
| Control Side *MF* - Control Side *S* \| Control Top *MF* - Control Top *S* | 3.154 | 0.207 | 15.232 | 0.000 | *** |
| Exclusion Side *MF* - Exclusion Side *S \|* Exclusion Top *MF* - Exclusion Top *S* | 3.154 | 0.207 | 15.232 | 0.000 | *** |
| *90 days* |  |  |  |  |  |
| Control Side *MF* - Control Side *S* \| Control Top *MF* - Control Top *S* | 3.154 | 0.207 | 15.232 | 0.000 | *** |
| Exclusion Side *MF* - Exclusion Side *S* | 3.154 | 0.207 | 15.232 | 0.000 | *** |
| *240 days* |  |  |  |  |  |
| Control Side *MF* - Control Side *S* \| Control Top *MF* - Control Top *S* | 3.154 | 0.207 | 15.232 | 0.000 | *** |
| Exclusion Side *MF* - Exclusion Side *S \|* Exclusion Top *MF* - Exclusion Top *S* | 3.154 | 0.207 | 15.232 | 0.000 | *** |
| **Model 2** | | | | | |
| **GLMM fit by maximum likelihood (Laplace Approximation)** | | | | | |
| Family: binomial (logit) |  |  |  |  |  |
| Formula: Survival ~ Site + DeviceType + LifeStage + (1/DeviceNumber) + (1 \| PlugPosition) | | | | | |
| No. Observations: 614  No. Groups: Plug Position, 2 |  |  |  |  |  |
|  | **estimate** | **std.err** | **z.value** | **p.value** | **signif.code** |
| (Intercept) | 0.921 | 0.290 | 3.180 | 0.002 | ** |
| Site2 | -0.076 | 0.296 | -0.255 | 0.798 |  |
| Site3 | -0.154 | 0.296 | -0.522 | 0.602 |  |
| Site4 | -0.092 | 0.303 | -0.303 | 0.762 |  |
| DeviceType Exclusion | 0.818 | 0.221 | 3.699 | 2.2e-04 | *** |
| DeviceType Cage | 2.105 | 0.536 | 3.928 | 8.6e-05 | *** |
| LifeStage spat | -3.412 | 0.385 | -8.855 | 2e-16 | *** |

**2.2. Coral grazing**

**Table S5.** Model output for coral grazing. Generalized linear mixed effects (GLMM; R Software) models were used with coral grazing by fishes as the response and device type (control and exclusion), plug position (top and side), survey timepoint (2, 90, and 240 days) and site (n=4) as predictors; the exact model used is defined in the table. Significance codes represent p values with “*” as <0.05, “**” as <0.01, and “***” as <0.001.

| **Grazing Model** | | | | | | |
| --- | --- | --- | --- | --- | --- | --- |
| **GLMM fit by maximum likelihood (Laplace Approximation)** | | | |  |  |  |
| Family: binomial (logit) | | |  | |  |  |
| Formula: Grazing ~ DeviceType * PlugPosition + LifeStage + Site + (1 \| DeviceNumber)  No. Observations: 380  No. Groups: Device Number, 149 | | | | | | |
|  | **estimate** | **std.err** | **z.value** | | **p.value** | **signif.code** |
| (Intercept) | -0.874 | 0.491 | -1.779 | | 0.075 |  |
| Device Type Exclusion | -2.722 | 0.570 | -4.777 | | 0.000 | *** |
| Plug Position Side | -0.830 | 0.393 | -2.113 | | 0.035 | * |
| Life Stage spat | 0.678 | 0.373 | 1.819 | | 0.069 |  |
| Site2 | -0.070 | 0.590 | -0.119 | | 0.905 |  |
| Site3 | 3.484 | 0.710 | 4.910 | | 0.000 | *** |
| Site4 | 1.031 | 0.586 | 1.761 | | 0.078 |  |
| Device Type Exclusion: Plug Position Side | -2.940 | 1.176 | -2.501 | | 0.012 | * |
| **Planned Comparisons** | | |  | |  |  |
| **Device Type** | |  |  | |  |  |
| *Results for coral spat* | **estimate** | **std.err** | **z.value** | | **p.value** | **signif.code** |
| Control Top - Exclusion Top | 2.722 | 0.570 | 4.777 | | 0.001 | ** |
| Control Side - Exclusion Side | 5.662 | 1.201 | 4.713 | | 0.001 | ** |
| *Results for coral microfragments* | **estimate** | **std.err** | **z.value** | | **p.value** | **signif.code** |
| Control Top - Exclusion Top | 2.722 | 0.570 | 4.777 | | 0.001 | ** |
| Control Side - Exclusion Side | 5.662 | 1.201 | 4.713 | | 0.001 | ** |
| **Site** |  |  |  | |  |  |
| *Results for coral spat* | **estimate** | **std.err** | **z.value** | | **p.value** | **signif.code** |
| Site 1 - Site 3 | -3.484 | 0.710 | -4.910 | | 0.000 | *** |
| Site 2 - Site 3 | -3.554 | 0.677 | -5.251 | | 0.000 | *** |
| Site 3 - Site 4 | 2.453 | 0.618 | 3.967 | | 0.026 | * |
| *Results for coral microfragments* | **estimate** | **std.err** | **z.value** | | **p.value** | **signif.code** |
| Site 1 - Site 3 | -3.484 | 0.710 | -4.910 | | 0.000 | *** |
| Site 2 - Site 3 | -3.554 | 0.677 | -5.251 | | 0.000 | *** |
| Site 3 – Site 4 | 2.453 | 0.618 | 3.967 | | 0.026 | * |

**2.3. Fish abundance and feeding**

**Table S6.** Model output for *in situ* fish abundance data. Linear and mixed effects models (lm, glm and glmmTMB; R Software) were used with fish abundance or PC1 as the response and fish family, site, and survey timepoint as the predictors. Models are defined in the table. Significance codes represent p-values with “*” as <0.05, “**” as <0.01, and “***” as <0.001.

| **Fish Abundance Models** | | | | | | | |
| --- | --- | --- | --- | --- | --- | --- | --- |
| **Linear Models** | | | | | | | |
| **PC1 ~ Site** | **estimate** | **std.err** | **statistic** | **cf.low** | **cf.high** | **p.value** | **signif.code** |
| (Intercept) | -0.425 | 0.128 | -3.325 | -0.681 | -0.169 | 0.002 | ** |
| Site2 | 0.777 | 0.181 | 4.298 | 0.415 | 1.139 | <0.001 | *** |
| Site3 | 0.648 | 0.168 | 3.847 | 0.310 | 0.986 | <0.001 | *** |
| Site4 | 0.157 | 0.188 | 0.836 | -0.220 | 0.534 | 0.407 |  |
| **PC1 ~ Labridae[Scarini]** | |  |  |  |  |  |  |
| (Intercept) | -0.304 | 0.169 | -1.799 | -0.642 | 0.034 | 0.077 |  |
| Labridae (Scarini) | 0.017 | 0.008 | 1.990 | 0.000 | 0.034 | 0.051 |  |
| **PC1 ~ Siganidae** | |  |  |  |  |  |  |
| (Intercept) | -0.011 | 0.093 | -0.117 | -0.196 | 0.175 | 0.907 |  |
| Siganidae | 0.010 | 0.052 | 0.196 | -0.095 | 0.115 | 0.845 |  |
| **PC1 ~ Acanthuridae** | |  |  |  |  |  |  |
| (Intercept) | 0.417 | 0.086 | 4.860 | 0.245 | 0.589 | <0.001 |  |
| Acanthuridae | -0.161 | 0.025 | -6.460 | -0.211 | -0.111 | <0.001 | *** |
| **PC1 ~ Chaetodonidae** | |  |  |  |  |  |  |
| (Intercept) | 0.601 | 0.102 | 5.878 | 0.396 | 0.806 | <0.001 |  |
| Chaetodonidae | -0.146 | 0.021 | -6.959 | -0.188 | -0.104 | <0.001 | *** |
| **PC1 ~ Pomacentridae** | |  |  |  |  |  |  |
| (Intercept) | -0.117 | 0.110 | -1.061 | -0.338 | 0.104 | 0.293 |  |
| Pomacentridae | 0.003 | 0.002 | 1.418 | -0.001 | 0.007 | 0.162 |  |
| **Generalized Linear Mixed Effects Model** | | | | | | | |
| Formula: Total_Count ~ Fish_Family + Site + Timepoint + (1 \| Replicate) | | | | | | |  |
| Family: truncated poisson (log) | | |  |  |  |  |  |
| No. observations: 354 | |  |  |  |  |  |  |
| Groups (Replicates): 11 | |  |  |  |  |  |  |
|  |  | **coefficient** | **std.error** | **z.value** | **p.value** | **signif.code** |  |
| (Intercept) | | 1.288 | 0.161 | 7.990 | <0.001 | *** |  |
| Balistidae | | -1.662 | 0.484 | -3.433 | 0.001 | *** |  |
| Chaetodontidae | | 0.261 | 0.110 | 2.365 | 0.018 | * |  |
| Labridae (Scarini) | | 1.713 | 0.093 | 18.428 | <0.001 | *** |  |
| Pomacentridae | | 2.523 | 0.090 | 28.003 | <0.001 | *** |  |
| Siganidae | | -0.612 | 0.184 | -3.327 | 0.001 | ** |  |
| Site 2 | | 0.026 | 0.045 | 0.561 | 0.575 |  |  |
| Site 3 | | -0.378 | 0.051 | -7.443 | <0.001 | *** |  |
| Site 4 | | 0.159 | 0.046 | 3.457 | 0.001 | ** |  |
| 90 days | | 0.055 | 0.038 | 1.446 | 0.148 |  |  |
| 240 days | | -0.605 | 0.048 | -12.646 | <0.001 | *** |  |
| **GLMM *Planned Comparisons*** | | | | | | | |
| ***Sites*** | | | **estimate** | **std.err** | **z.value** | **p.value** | **signif.code** |
| Site 1 – Site 3 (*All families*) | | | 0.378 | 0.051 | 7.443 | <0.001 | *** |
| Site 2 – Site 3 (*All families*) | | | 0.404 | 0.051 | 7.938 | <0.001 | *** |
| Site 3 – Site 4 (*All families*) | | | -0.537 | 0.051 | -10.488 | <0.001 | *** |

**Table S7.** Model output for fish feeding (bite) data. Linear and mixed effects models (lm, glm and glmmTMB; R Software) were used with total bites or PC1 as the response and fish family or fish species, site, and survey timepoint as the predictors. Models are defined in the table. Significance codes represent p values with “*” as <0.05, “**” as <0.01, and “***” as <0.001.

| **Fish Feeding Models** | | | | | | | |
| --- | --- | --- | --- | --- | --- | --- | --- |
| **Linear Models** | | | | | | | |
| **PC1~Site** | **estimate** | **std.err** | **statistic** | **cf.low** | **cf.high** | **p.value** | **signif.code** |
| (Intercept) | -0.181 | 0.090 | -2.004 | -0.360 | -0.002 | 0.047 | ***** |
| Site2 | 0.505 | 0.128 | 3.949 | 0.252 | 0.758 | <0.001 | *** |
| Site3 | 0.110 | 0.128 | 0.858 | -0.143 | 0.363 | 0.392 |  |
| Site4 | 0.114 | 0.126 | 0.905 | -0.135 | 0.363 | 0.367 |  |
| **PC1 ~ *Scarus globiceps*** | | | |  |  |  |  |
| (Intercept) | -0.202 | 0.034 | -6.037 | -0.269 | -0.136 | <0.001 | ******* |
| *Scarus_globiceps* | 0.002 | 0.000 | 13.944 | 0.002 | 0.003 | <0.001 | ******* |
| **PC1 ~ *Scarus schlegeli*** | | |  |  |  |  |  |
| (Intercept) | -0.146 | 0.025 | -5.751 | -0.196 | -0.096 | <0.001 | ******* |
| *Scarus_schlegeli* | 0.008 | 0.000 | 19.415 | 0.007 | 0.009 | <0.001 | ******* |
| **PC1 ~ *Chlorurus spirulus*** | | | |  |  |  |  |
| (Intercept) | -0.162 | 0.041 | -3.950 | -0.243 | -0.081 | <0.001 | ******* |
| *Chlorurus_spirulus* | 0.011 | 0.001 | 9.246 | 0.008 | 0.013 | <0.001 | ******* |
| **PC1 ~ *Scarus dimidiatus*** | | | |  |  |  |  |
| (Intercept) | 0.002 | 0.048 | 0.043 | -0.092 | 0.097 | 0.966 |  |
| *Scarus_dimidiatus* | -0.004 | 0.009 | -0.391 | -0.022 | 0.015 | 0.696 |  |
| **Generalized Linear Mixed Effects Model (glmmTMB)** | | | | | | | |
| **Model – *Parrotfish species*** | | | | | | | |
| Family: truncated_poisson (log) | | | |  |  |  |  |
| Formula: TotalBites ~ Species + Site + (1 \| Video_Name) | | | | | | |  |
| Zero inflation: ~1  Number of observations: 8040  Number of groups: 134 | | |  |  |  |  |  |
|  |  |  | **estimate** | **std.err** | **z.value** | **p.value** | **signif.code** |
| (Intercept) | | | 1.486 | 0.261 | 5.704 | <0.001 | *** |
| *Chlorurus_microrhinos* | | | -0.128 | 0.091 | -1.405 | 0.160 |  |
| *Chlorurus_spirulus* | | | 0.366 | 0.078 | 4.690 | <0.001 | *** |
| *Scarus_altipinnis* | | | -2.431 | 0.283 | -8.579 | <0.001 | *** |
| *Scarus_chameleon* | | | -3.467 | 0.933 | -3.715 | <0.001 | *** |
| *Scarus_dimidiatus* | | | 1.700 | 0.161 | 10.546 | <0.001 | *** |
| *Scarus_flavipectoralis* | | | -0.508 | 0.089 | -5.734 | <0.001 | *** |
| *Scarus_frenatus* | | | -0.246 | 0.126 | -1.952 | 0.051 |  |
| *Scarus_globiceps* | | | 1.939 | 0.075 | 25.719 | <0.001 | *** |
| *Scarus_niger* | | | 0.385 | 0.081 | 4.785 | <0.001 | *** |
| *Scarus_oviceps* | | | 0.603 | 0.390 | 1.546 | 0.122 |  |
| *Scarus_rubroviolaceus* | | | -0.984 | 0.653 | -1.507 | 0.132 |  |
| *Scarus_schlegeli* | | | 0.953 | 0.078 | 12.183 | <0.001 | *** |
| *Scarus_spinus* | | | 0.854 | 0.094 | 9.059 | <0.001 | *** |
| *Scarus_spp. unk* | | | 0.707 | 0.114 | 6.186 | <0.001 | *** |
| Site2 | | | 1.384 | 0.313 | 4.426 | <0.001 | *** |
| Site3 | | | 1.001 | 0.326 | 3.072 | 0.002 | ** |
| Site4 | | | 0.874 | 0.314 | 2.783 | 0.005 | ** |
| **Planned Comparisons** | | | | | | | |
| Site 1 - Site 2 | | | **Estimate** | **Std.Error** | **z.value** | **p.value** | **signif.code** |
| Parrotfish species *(Chlorurus bleekeri, C. microrhinos, C. spirulus, Scarus chameleon, S. dimidiatus, S. flavipectoralis, S. frenatus, S. niger, S. oviceps, S. rubroviolaceus, S. schlegeli, S. spinus)* | | | -1.384 | 0.313 | -4.426 | 0.013 | * |

**Table S8.** Observed bite data from GoPro Video footage. The table includes the number of observations (bites) separated by feeding category, functional group, and species by site. A total of 33-35 video clips (397 minutes of footage) per site were used from two time points (2 and 240 days).

| **Feeding category** | **Functional group and species** | **Number of observations by site** | | | |
| --- | --- | --- | --- | --- | --- |
|  |  | **Feeding (Bites)** | | | |
|  |  | **Site 1** | **Site 2** | **Site 3** | **Site 4** |
| **Herbivores** | **Scraper** | **751** | **7363** | **3768** | **3779** |
|  | *Scarus globiceps* | 492 | 4720 | 2765 | 3134 |
|  | *Scarus rubroviolaceus* | 0 | 0 | 3 | 0 |
|  | *Scarus niger* | 85 | 171 | 415 | 415 |
|  | *Scarus chameleon* | 0 | 0 | 2 | 1 |
|  | *Scarus flavipectoralis* | 0 | 392 | 0 | 42 |
|  | *Scarus altipinnis* | 0 | 23 | 0 | 0 |
|  | *Scarus oviceps* | 8 | 0 | 0 | 0 |
|  | *Scarus dimidiatus* | 0 | 0 | 7 | 70 |
|  | *Scarus frenatus* | 7 | 96 | 5 | 54 |
|  | *Scarus schlegeli* | 46 | 1771 | 505 | 29 |
|  | *Scarus spinus* | 113 | 190 | 66 | 34 |
|  | **Excavator** | **170** | **1117** | **508** | **880** |
|  | *Scarus rubroviolaceus* | 0 | 0 | 3 | 0 |
|  | *Chlorurus bleekeri* | 0 | 67 | 98 | 55 |
|  | *Scarus altipinnis* | 0 | 23 | 0 | 0 |
|  | *Chlorurus microrhinos* | 2 | 319 | 70 | 15 |
|  | *Chlorurus spirulus* | 168 | 708 | 337 | 810 |
|  | **Cropper** | **20** | **148** | **94** | **38** |
|  | *Acanthurus nicrofuscus* | 0 | 0 | 42 | 0 |
|  | *Siganus punctatus* | 0 | 104 | 0 | 0 |
|  | *Siganus corallinus* | 1 | 17 | 1 | 1 |
|  | *Siganus vulpinus* | 19 | 8 | 0 | 0 |
|  | *Stegastes apicalis* | 0 | 19 | 0 | 23 |
|  | *Chrysiptera rex* | 0 | 0 | 0 | 14 |
|  | *Escenius mandibularis* | 0 | 0 | 51 | 0 |
|  | **Browser** | **2** | **4** | **0** | **0** |
|  | *Naso unicornis* | 2 | 4 | 0 | 0 |
|  | **Brusher** | **192** | **132** | **341** | **450** |
|  | *Ctenochaetus striatus* | 192 | 132 | 341 | 450 |
| **Corallivores** | *Chaetodon baronessa* | 10 | 32 | 31 | 12 |
|  | *Chaetodon citrinellus* | 34 | 19 | 0 | 3 |
|  | *Chaetodon trifasciatus* | 20 | 4 | 20 | 14 |
|  | *Chaetodon rainfordi* | 64 | 44 | 9 | 35 |
|  | *Chaetodon vagabondus* | 8 | 0 | 0 | 0 |
|  | Labrichthys unilineatus | 0 | 0 | 0 | 9 |
|  | *Escenius stictus* | 0 | 0 | 15 | 0 |
| **Omnivores** | *Centropyge vrolikii* | 14 | 0 | 0 | 0 |
|  | *Pomacentrus bankanensis* | 301 | 174 | 380 | 320 |
|  | *Amblygobius spp.* | 0 | 20 | 0 | 0 |
|  | *Pomacentrus coelestis* | 0 | 15 | 0 | 0 |
|  | *Amblygobius phalaena* | 0 | 0 | 0 | 4 |
| **Carnivores** | *Epinephelus merra* | 2 | 0 | 0 | 0 |
|  | *Gomphosus varius* | 2 | 0 | 3 | 0 |
|  | *Cheilinus chlorourus* | 3 | 0 | 0 | 0 |
|  | *Halichoeres hortulanus* | 1 | 0 | 0 | 0 |
|  | *Halichoeres marginatus* | 9 | 0 | 0 | 16 |
|  | *Hemigymnus fasciatus* | 5 | 0 | 3 | 1 |
|  | *Hemigymnus melapterus* | 3 | 22 | 7 | 5 |
|  | *Thalassoma nigrofasciatum* | 13 | 46 | 18 | 12 |
|  | *Sufflamen chrysopterum* | 19 | 0 | 7 | 6 |
|  | *Cheilinus fasciatus* | 0 | 4 | 1 | 0 |
|  | *Thalassoma hardwicke* | 0 | 2 | 12 | 0 |
|  | *Epibulus insidiator* | 0 | 9 | 0 | 0 |
|  | *Halichoeres margaritaceus* | 0 | 55 | 0 | 0 |
|  | *Halichoeres melanurus* | 0 | 6 | 2 | 0 |
|  | *Parupeneus bifasciatus* | 0 | 5 | 3 | 0 |
|  | *Parupeneus multifasciatus* | 0 | 0 | 0 | 4 |
|  | *Pomacanthus sextriatus* | 0 | 3 | 6 | 0 |
|  | *Stethojulis bandanensis* | 0 | 8 | 4 | 25 |
|  | *Chelmon rostratus* | 0 | 0 | 0 | 1 |
|  | *Thalassoma lunare* | 0 | 1 | 0 | 0 |
|  | *Coris gaimard* | 0 | 0 | 1 | 0 |
|  | *Halichoeres nebulosus* | 0 | 0 | 18 | 6 |
|  | *Oxycheilinus digrammus* | 0 | 0 | 1 | 0 |
|  | *Coris aurilineata* | 0 | 0 | 0 | 9 |
| **Total bites** |  | **1643** | **9233** | **5252** | **5629** |
| **Family color codes** | | |  |  |  |
|  | Labridae | Wrasse |  |  |  |
|  | Labridae (Scarini) | Parrotfishes |  |  |  |
|  | Blennidae | Blennies |  |  |  |
|  | Pomacentridae | Damselfishes |  |  |  |
|  | Balistidae | Triggerfishes |  |  |  |
|  | Gobiidae | Gobbies |  |  |  |
|  | Mullidae | Goatfishes |  |  |  |
|  | Pomacanthiidae | Angelfishes |  |  |  |
|  | Chaetodontidae | Butterflyfishes |  |  |  |
|  | Serranidae | Groupers, Rockcods | |  |  |
|  | Acanthuridae | Surgeonfishes |  |  |  |
|  | Siganidae | Rabbitfishes |  |  |  |

**2.4. Benthic composition**

**Table S9:** Model output for benthic data. Linear models (lm aand glm; R Software) were used to identify relationships between the response and the predictors. First, we use PC1 as the response with abundance and site as the predictors. Next, we use abundance (percent cover) as the response and site as the predictor. Lastly, coral survival and grazing were used as the response variables with the interaction between site and abundance as predictors. Significance codes represent p values, with “.” as <0.1, “*” as <0.05, “**” as <0.01, and “***” as <0.001.

| **Benthic Composition Models** | | | | | | | |
| --- | --- | --- | --- | --- | --- | --- | --- |
| **Percent Abundance data** | | | | | | | |
| **Linear Models** | **estimate** | **std.err** | **statistic** | **cf.low** | **cf.high** | **p.value** | **signif.code** |
| **PC1 ~ Site** |  |  |  |  |  |  |  |
| (Intercept) | -0.196 | 0.158 | -1.238 | -0.512 | 0.120 | 0.220 |  |
| Site 2 | 0.659 | 0.233 | 2.831 | 0.195 | 1.123 | 0.006 | ** |
| Site 3 | 0.122 | 0.236 | 0.518 | -0.348 | 0.593 | 0.606 |  |
| Site 4 | 0.030 | 0.233 | 0.131 | -0.433 | 0.494 | 0.896 |  |
| **PC1 ~ Cyanobacteria** |  |  |  |  |  |  |  |
| (Intercept) | -0.106 | 0.091 | -1.156 | -0.287 | 0.076 | 0.251 |  |
| Cyanobacteria | 0.020 | 0.007 | 2.926 | 0.006 | 0.033 | 0.005 | ** |
| **PC1 ~ Octocorals** |  |  |  |  |  |  |  |
| (Intercept) | -0.071 | 0.078 | -0.911 | -0.226 | 0.084 | 0.365 |  |
| Octocorals | 0.296 | 0.059 | 5.052 | 0.179 | 0.413 | <0.001 | *** |
| **PC1 ~ *Pocillopora*** |  |  |  |  |  |  |  |
| (Intercept) | -0.079 | 0.039 | -2.028 | -0.226 | 0.084 | 0.046 | * |
| Pocillopora | 1.418 | 0.079 | 17.914 | 0.179 | 0.413 | <0.001 | *** |
| **PC1 ~ *Dipsastrea*** |  |  |  |  |  |  |  |
| (Intercept) | -0.079 | 0.039 | -2.028 | -0.157 | -0.001 | 0.046 | * |
| Dipsastrea | 0.473 | 0.026 | 17.914 | 1.261 | 1.576 | <0.001 | *** |
| **PC1 ~ Turf algae** |  |  |  |  |  |  |  |
| (Intercept) | 0.383 | 0.199 | 1.923 | -0.014 | 0.779 | 0.058 | . |
| Turf algae | -0.010 | 0.005 | -2.133 | -0.020 | -0.001 | 0.036 | * |
| **PC1 ~ *Acropora* digitate** |  |  |  |  |  |  |  |
| (Intercept) | 0.004 | 0.112 | 0.035 | -0.219 | 0.227 | 0.972 |  |
| Acropora digitate | -0.001 | 0.018 | -0.058 | -0.038 | 0.036 | 0.954 |  |
| **PC1 ~ Hard rock** |  |  |  |  |  |  |  |
| (Intercept) | 0.052 | 0.095 | 0.552 | -0.137 | 0.241 | 0.583 |  |
| Hard rock | -0.026 | 0.018 | -1.415 | -0.062 | 0.010 | 0.161 |  |
| **PC1 ~ Rubble** |  |  |  |  |  |  |  |
| (Intercept) | 0.011 | 0.089 | 0.122 | -0.167 | 0.189 | 0.903 |  |
| Rubble | -0.069 | 0.087 | -0.795 | -0.241 | 0.104 | 0.429 |  |
| **Cyanobacteria ~ Site** |  |  |  |  |  |  |  |
| (Intercept) | 4.048 | 2.354 | 1.719 | -0.643 | 8.739 | 0.090 | . |
| Site 2 | 12.050 | 3.458 | 3.484 | 5.160 | 18.941 | 0.001 | ** |
| Site 3 | -3.585 | 3.509 | -1.022 | -10.578 | 3.408 | 0.310 |  |
| Site 4 | -3.296 | 3.458 | -0.953 | -10.186 | 3.595 | 0.344 |  |
| **Octocorals ~ Site** |  |  |  |  |  |  |  |
| (Intercept) | 0.000 | 0.278 | 0.000 | -0.553 | 0.553 | 1.000 |  |
| Site 2 | 0.229 | 0.408 | 0.561 | -0.583 | 1.041 | 0.576 |  |
| Site 3 | 0.797 | 0.414 | 1.927 | -0.027 | 1.621 | 0.058 | . |
| Site 4 | 0.000 | 0.408 | 0.000 | -0.812 | 0.812 | 1.000 |  |
| ***Pocillopora* ~ Site** |  |  |  |  |  |  |  |
| (Intercept) | 0.000 | 0.105 | 0.000 | -0.209 | 0.209 | 1.000 |  |
| Site 2 | 0.229 | 0.154 | 1.485 | -0.078 | 0.536 | 0.142 |  |
| Site 3 | 0.000 | 0.156 | 0.000 | -0.312 | 0.312 | 1.000 |  |
| Site 4 | 0.000 | 0.154 | 0.000 | -0.307 | 0.307 | 1.000 |  |
| ***Dipsastrea* ~ Site** |  |  |  |  |  |  |  |
| (Intercept) | 0.000 | 0.315 | 0.000 | -0.627 | 0.627 | 1.000 |  |
| Site 2 | 0.686 | 0.462 | 1.485 | -0.234 | 1.607 | 0.142 |  |
| Site 3 | 0.000 | 0.469 | 0.000 | -0.935 | 0.935 | 1.000 |  |
| Site 4 | 0.000 | 0.462 | 0.000 | -0.921 | 0.921 | 1.000 |  |
| **Turf algae ~ Site** |  |  |  |  |  |  |  |
| (Intercept) | 39.455 | 3.754 | 10.509 | 31.974 | 46.935 | <0.001 | *** |
| Site 2 | -10.343 | 5.515 | -1.875 | -21.332 | 0.646 | 0.065 | . |
| Site 3 | 0.584 | 5.597 | 0.104 | -10.568 | 11.736 | 0.917 |  |
| Site 4 | -0.224 | 5.515 | -0.041 | -11.214 | 10.765 | 0.968 |  |
| **Coralline algae ~ Site** |  |  |  |  |  |  |  |
| (Intercept) | 31.510 | 3.977 | 7.923 | 23.585 | 39.434 | <0.001 | *** |
| Site 2 | 0.377 | 5.842 | 0.065 | -11.264 | 12.018 | 0.949 |  |
| Site 3 | 4.193 | 5.929 | 0.707 | -7.621 | 16.006 | 0.482 |  |
| Site 4 | 4.542 | 5.842 | 0.777 | -7.099 | 16.182 | 0.439 |  |
| **Harl rock ~ Site** |  |  |  |  |  |  |  |
| (Intercept) | 5.186 | 0.954 | 5.435 | 3.285 | 7.088 | <0.001 | *** |
| Site 2 | -4.180 | 1.402 | -2.982 | -6.973 | -1.387 | 0.004 | ** |
| Site 3 | -4.008 | 1.423 | -2.817 | -6.842 | -1.173 | 0.006 | ** |
| Site 4 | -4.967 | 1.402 | -3.543 | -7.760 | -2.174 | <0.001 | *** |
| **Rubble ~ Site** |  |  |  |  |  |  |  |
| (Intercept) | 0.198 | 0.220 | 0.899 | -0.240 | 0.636 | 0.372 |  |
| Site 2 | -0.198 | 0.323 | -0.612 | -0.841 | 0.446 | 0.542 |  |
| Site 3 | -0.198 | 0.328 | -0.603 | -0.851 | 0.455 | 0.548 |  |
| Site 4 | 0.223 | 0.323 | 0.692 | -0.420 | 0.867 | 0.491 |  |
| ***Acropora* digitate ~ Site** |  |  |  |  |  |  |  |
| (Intercept) | 4.422 | 1.045 | 4.232 | 2.340 | 6.504 | <0.001 | *** |
| Site 2 | -0.952 | 1.535 | -0.620 | -4.011 | 2.106 | 0.537 |  |
| Site 3 | -0.648 | 1.558 | -0.416 | -3.752 | 2.456 | 0.679 |  |
| Site 4 | -1.245 | 1.535 | -0.811 | -4.304 | 1.813 | 0.420 |  |
| **Planned Comparisons** | | | | | | | |
|  |  | | **estimate** | **etd.err** | **t.value** | **p.value** | **signif.code** |
| **Cyanobacteria** | Site 2 – Site 4 | | 15.346 | 3.583 | 4.283 | <0.001 | *** |
|  | Site 2 – Site 1 | | 12.050 | 3.458 | 3.484 | <0.001 | *** |
|  | Site 2 – Site 3 | | 15.635 | 3.632 | 4.305 | <0.001 | *** |
| **Octocorals** | Site 3 – Site 4 | | 0.797 | 0.428 | 1.862 | 0.067 | . |
|  | Site 3 – Site 1 | | 0.797 | 0.414 | 1.927 | 0.058 | . |
|  | Site 3 – Site 2 | | 0.568 | 0.428 | 1.327 | 0.188 |  |
| **Turf Algae** | Site 2 – Site 4 | | -10.118 | 5.713 | -1.771 | 0.081 | . |
|  | Site 2 – Site 1 | | -10.343 | 5.515 | -1.875 | 0.065 | . |
|  | Site 2 – Site 3 | | -10.927 | 5.792 | -1.886 | 0.063 | . |
| **Harf Rock** | Site 2 – Site 4 | | 0.787 | 1.452 | 0.542 | 0.590 | . |
|  | Site 2 – Site 1 | | -4.180 | 1.402 | -2.982 | 0.004 | ** |
|  | Site 2 – Site 3 | | -0.173 | 1.472 | -0.117 | 0.907 |  |
|  | Site 3 – Site 4 | | 0.959 | 1.472 | 0.651 | 0.517 |  |
|  | Site 3 – Site 1 | | -4.008 | 1.423 | -2.817 | 0.006 | ** |
|  | Site 4 – Site 1 | | -4.967 | 1.402 | -3.543 | <0.001 | *** |
| **Coral Survival** |  |  |  |  |  |  |  |
| **Linear Models** | **estimate** | **std.err** | **statistic** | **cf.low** | **cf.high** | **p.value** | **signif.code** |
| *Coral Spat* | | | | | | | |
| **Survival ~ Cyanobacteria * Site** | | | | | | | |
| (Intercept) | 0.346 | 0.112 | 3.099 | 0.123 | 0.569 | 0.003 | ** |
| CYAN | -0.007 | 0.015 | -0.471 | -0.036 | 0.022 | 0.639 |  |
| Site 2 | 0.194 | 0.171 | 1.135 | -0.147 | 0.535 | 0.260 |  |
| Site 3 | -0.346 | 0.155 | -2.233 | -0.655 | -0.037 | 0.029 | * |
| Site 4 | 0.114 | 0.156 | 0.730 | -0.197 | 0.425 | 0.468 |  |
| CYAN: Site 2 | 0.003 | 0.016 | 0.180 | -0.028 | 0.034 | 0.858 |  |
| CYAN: Site 3 | 0.007 | 0.057 | 0.122 | -0.106 | 0.120 | 0.903 |  |
| CYAN: Site 4 | -0.045 | 0.053 | -0.843 | -0.151 | 0.061 | 0.402 |  |
| **Survival ~ Hard Rock * Site** | | | | | | | |
| (Intercept) | 0.268 | 0.113 | 2.375 | 0.043 | 0.494 | 0.020 | * |
| ROCK | 0.010 | 0.013 | 0.759 | -0.016 | 0.035 | 0.450 |  |
| Site 2 | 0.275 | 0.155 | 1.775 | -0.034 | 0.584 | 0.080 | . |
| Site 3 | -0.268 | 0.157 | -1.709 | -0.582 | 0.045 | 0.092 | . |
| Site 4 | 0.120 | 0.152 | 0.792 | -0.183 | 0.424 | 0.431 |  |
| ROCK: Site 2 | -0.079 | 0.040 | -1.984 | -0.159 | 0.000 | 0.051 | * |
| ROCK: Site 3 | -0.010 | 0.036 | -0.270 | -0.080 | 0.061 | 0.788 |  |
| ROCK: Site 4 | 0.137 | 0.107 | 1.278 | -0.077 | 0.351 | 0.205 |  |
| **Survival ~ *Acropora* digitate * site** | | | | | | | |
| (Intercept) | 0.150 | 0.117 | 1.281 | -0.084 | 0.384 | 0.204 |  |
| ACD | 0.038 | 0.016 | 2.302 | 0.005 | 0.071 | 0.024 | * |
| Site 2 | 0.399 | 0.179 | 2.224 | 0.041 | 0.756 | 0.029 | * |
| Site 3 | -0.150 | 0.168 | -0.896 | -0.485 | 0.184 | 0.374 |  |
| Site 4 | 0.279 | 0.185 | 1.511 | -0.089 | 0.648 | 0.135 |  |
| ACD: Site 2 | -0.060 | 0.031 | -1.904 | -0.122 | 0.003 | 0.061 | . |
| ACD: Site 3 | -0.038 | 0.024 | -1.609 | -0.085 | 0.009 | 0.112 |  |
| ACD: Site 4 | -0.041 | 0.036 | -1.118 | -0.113 | 0.032 | 0.267 |  |
| *Coral Microfragments* | | | | | | | |
| **Survival ~ Cyanobacteria * Site** | | | | | | | |
| (Intercept) | 1.000 | 0.067 | 14.876 | 0.866 | 1.134 | 0.000 | *** |
| CYAN | 0.000 | 0.009 | 0.000 | -0.018 | 0.018 | 1.000 |  |
| Site 2 | -0.247 | 0.103 | -2.401 | -0.452 | -0.042 | 0.019 | * |
| Site 3 | -0.059 | 0.093 | -0.630 | -0.245 | 0.127 | 0.530 |  |
| Site 4 | -0.120 | 0.094 | -1.283 | -0.307 | 0.067 | 0.204 |  |
| CYAN: Site 2 | 0.006 | 0.009 | 0.593 | -0.013 | 0.024 | 0.555 |  |
| CYAN: Site 3 | 0.007 | 0.034 | 0.207 | -0.061 | 0.075 | 0.837 |  |
| CYAN: Site 4 | 0.020 | 0.032 | 0.625 | -0.044 | 0.084 | 0.534 |  |
| **Survival ~ Hard Rock * Site** | | | | | | | |
| (Intercept) | 1.000 | 0.071 | 14.058 | 0.858 | 1.142 | <0.001 | *** |
| H_ROCK | 0.000 | 0.008 | 0.000 | -0.016 | 0.016 | 1.000 |  |
| Site 2 | -0.181 | 0.098 | -1.857 | -0.376 | 0.013 | 0.068 | . |
| Site 3 | -0.064 | 0.099 | -0.645 | -0.261 | 0.133 | 0.521 |  |
| Site 4 | -0.111 | 0.096 | -1.161 | -0.302 | 0.080 | 0.250 |  |
| ROCK: Site 2 | 0.023 | 0.025 | 0.923 | -0.027 | 0.073 | 0.359 |  |
| ROCK: Site 3 | 0.007 | 0.022 | 0.311 | -0.038 | 0.052 | 0.756 |  |
| ROCK: Site 4 | 0.027 | 0.067 | 0.395 | -0.108 | 0.161 | 0.694 |  |
| **Survival ~ *Acropora* digitate * site** | | | | | | | |
| (Intercept) | 1.000 | 0.072 | 13.971 | 0.857 | 1.143 | <0.001 | *** |
| ACD | 0.000 | 0.010 | 0.000 | -0.020 | 0.020 | 1.000 |  |
| Site 2 | -0.059 | 0.109 | -0.543 | -0.277 | 0.159 | 0.589 |  |
| Site 3 | -0.052 | 0.102 | -0.510 | -0.256 | 0.152 | 0.611 |  |
| Site 4 | 0.002 | 0.113 | 0.020 | -0.223 | 0.227 | 0.984 |  |
| ACD: Site 2 | -0.028 | 0.019 | -1.487 | -0.067 | 0.010 | 0.142 |  |
| ACD: Site 3 | -0.001 | 0.014 | -0.061 | -0.030 | 0.028 | 0.952 |  |
| ACD: Site 4 | -0.034 | 0.022 | -1.526 | -0.078 | 0.010 | 0.132 |  |
| **Survival ~ Turf algae * Site** | | | | | | | |
| (Intercept) | 1.000 | 0.155 | 6.455 | 0.691 | 1.309 | <0.001 | *** |
| TAL | 0.000 | 0.004 | 0.000 | -0.007 | 0.007 | 1.000 |  |
| Site 2 | -0.352 | 0.197 | -1.785 | -0.746 | 0.041 | 0.079 | . |
| Site 3 | -0.063 | 0.223 | -0.281 | -0.507 | 0.382 | 0.779 |  |
| Site 4 | 0.076 | 0.205 | 0.372 | -0.333 | 0.485 | 0.711 |  |
| TAL: Site 2 | 0.007 | 0.005 | 1.292 | -0.004 | 0.017 | 0.201 |  |
| TAL: Site 3 | 0.000 | 0.005 | 0.034 | -0.010 | 0.011 | 0.973 |  |
| TAL: Site 4 | -0.005 | 0.005 | -0.970 | -0.014 | 0.005 | 0.335 |  |
| **Survival ~ Coralline algae * Site** | | | | | | | |
| (Intercept) | 1.000 | 0.113 | 8.865 | 0.775 | 1.225 | <0.001 | *** |
| CCA | 0.000 | 0.003 | 0.000 | -0.006 | 0.006 | 1.000 |  |
| Site 2 | 0.106 | 0.152 | 0.695 | -0.198 | 0.409 | 0.489 |  |
| Site 3 | 0.007 | 0.185 | 0.038 | -0.363 | 0.377 | 0.970 |  |
| Site 4 | -0.319 | 0.173 | -1.846 | -0.665 | 0.026 | 0.069 | . |
| CCA: Site 2 | -0.008 | 0.004 | -2.011 | -0.016 | 0.000 | 0.048 | * |
| CCA: Site 3 | -0.002 | 0.005 | -0.357 | -0.012 | 0.008 | 0.722 |  |
| CCA: Site 4 | 0.006 | 0.005 | 1.308 | -0.003 | 0.015 | 0.195 |  |
| **Planned Comparisons** | | | | | | | |
|  |  | **site** | **estimate** | **etd.err** | **t.value** | **p.value** | **signif.code** |
| **Spat Survival ~ Cyano * Site** | | S2 – S1 | 0.194 | 0.171 | 1.135 | 0.642 |  |
|  |  | S3 – S1 | -0.346 | 0.155 | -2.233 | 0.111 |  |
|  |  | S4 – S1 | 0.114 | 0.156 | 0.730 | 0.871 |  |
|  |  | S3 – S2 | -0.540 | 0.168 | -3.211 | 0.009 | ** |
|  |  | S4 – S2 | 0.460 | 0.153 | 3.008 | 0.016 | * |
| **Spat Survival ~ Hard Rock * Site** | | S2 – S1 | 0.243 | 0.092 | 2.637 | 0.036 | * |
|  |  | S3 – S1 | -0.300 | 0.094 | -3.194 | 0.008 | ** |
|  |  | S4 – S1 | 0.089 | 0.090 | 0.987 | 0.679 |  |
|  |  | S3 – S2 | -0.544 | 0.152 | -3.573 | 0.003 | ** |
|  |  | S4 – S2 | 0.389 | 0.149 | 2.607 | 0.038 | * |
| **Spat Survival ~ *Acropora* dig. * Site** | | S2 – S1 | 0.267 | 0.116 | 2.308 | 0.077 | . |
|  |  | S3 – S1 | -0.282 | 0.107 | -2.645 | 0.034 | * |
|  |  | S4 – S1 | 0.147 | 0.120 | 1.229 | 0.512 |  |
|  |  | S3 – S2 | -0.549 | 0.181 | -3.034 | 0.012 | * |
|  |  | S4 – S2 | 0.430 | 0.187 | 2.303 | 0.078 | . |
| **Frag Survival ~ Cyano* Site** | | S2 – S1 | -0.247 | 0.103 | -2.401 | 0.076 | . |
|  |  | S3 – S1 | -0.059 | 0.093 | -0.630 | 0.912 |  |
|  |  | S4 – S1 | -0.120 | 0.094 | -1.283 | 0.548 |  |
|  |  | S3 – S2 | 0.188 | 0.101 | 1.859 | 0.233 |  |
|  |  | S4 – S2 | -0.062 | 0.092 | -0.668 | 0.898 |  |
| **Fish Grazing** | | | | | | | |
| **Linear Models** | **estimate** | **std.err** | **statistic** | **cf.low** | **cf.high** | **p.value** | **signif.code** |
| *Coral Spat* | | | | | | | |
| **Grazing ~ Cyanobacteria * Site** | | | | | | | |
| (Intercept) | 0.505 | 0.110 | 4.571 | 0.285 | 0.725 | <0.001 | *** |
| CYAN | -0.001 | 0.015 | -0.086 | -0.030 | 0.028 | 0.932 |  |
| Site 2 | -0.317 | 0.169 | -1.874 | -0.654 | 0.020 | 0.065 | . |
| Site 3 | 0.495 | 0.153 | 3.227 | 0.189 | 0.801 | 0.002 | ** |
| Site 4 | -0.025 | 0.154 | -0.164 | -0.333 | 0.282 | 0.871 |  |
| CYAN: Site 2 | 0.006 | 0.015 | 0.384 | -0.025 | 0.037 | 0.702 |  |
| CYAN: Site 3 | 0.001 | 0.056 | 0.022 | -0.111 | 0.113 | 0.982 |  |
| CYAN: Site 4 | 0.063 | 0.053 | 1.198 | -0.042 | 0.168 | 0.235 |  |
| **Grazing ~ Hard Rock * Site** | | | | | | | |
| (Intercept) | 0.642 | 0.111 | 5.796 | 0.421 | 0.864 | <0.001 | *** |
| ROCK | -0.027 | 0.012 | -2.216 | -0.052 | -0.003 | 0.030 | * |
| Site 2 | -0.340 | 0.152 | -2.239 | -0.644 | -0.037 | 0.028 | * |
| Site 3 | 0.358 | 0.154 | 2.321 | 0.050 | 0.665 | 0.023 | * |
| Site 4 | -0.087 | 0.149 | -0.583 | -0.384 | 0.211 | 0.562 |  |
| ROCK: Site 2 | -0.011 | 0.039 | -0.285 | -0.089 | 0.067 | 0.776 |  |
| ROCK: Site 3 | 0.027 | 0.035 | 0.789 | -0.042 | 0.097 | 0.433 |  |
| ROCK: Site 4 | -0.106 | 0.105 | -1.007 | -0.316 | 0.104 | 0.318 |  |
| **Grazing ~ *Acropora* digitate * Site** | | | | | | | |
| (Intercept) | 0.602 | 0.110 | 5.468 | 0.382 | 0.822 | <0.001 | *** |
| ACD | -0.023 | 0.015 | -1.492 | -0.054 | 0.008 | 0.140 |  |
| Site 2 | -0.523 | 0.168 | -3.113 | -0.859 | -0.188 | 0.003 | ** |
| Site 3 | 0.398 | 0.157 | 2.527 | 0.084 | 0.712 | 0.014 | * |
| Site 4 | -0.353 | 0.173 | -2.033 | -0.699 | -0.007 | 0.046 | * |
| ACD: Site 2 | 0.076 | 0.029 | 2.595 | 0.018 | 0.135 | 0.012 | * |
| ACD: Site 3 | 0.023 | 0.022 | 1.043 | -0.021 | 0.067 | 0.301 |  |
| ACD: Site 4 | 0.110 | 0.034 | 3.232 | 0.042 | 0.178 | 0.002 | ** |
| *Coral Microfragments* |  |  |  |  |  |  |  |
| **Grazing~ Cyanobacteria * Site** | |  |  |  |  |  |  |
| (Intercept) | 0.390 | 0.113 | 3.455 | 0.165 | 0.616 | <0.001 | *** |
| CYAN | -0.018 | 0.015 | -1.198 | -0.047 | 0.012 | 0.235 |  |
| Site 2 | 0.067 | 0.173 | 0.388 | -0.278 | 0.412 | 0.699 |  |
| Site 3 | 0.551 | 0.157 | 3.514 | 0.238 | 0.864 | <0.001 | *** |
| Site 4 | 0.189 | 0.158 | 1.196 | -0.126 | 0.503 | 0.236 |  |
| CYAN: Site 2 | 0.022 | 0.016 | 1.408 | -0.009 | 0.053 | 0.164 |  |
| CYAN: Site 3 | 0.025 | 0.057 | 0.434 | -0.089 | 0.139 | 0.666 |  |
| CYAN: Site 4 | 0.088 | 0.054 | 1.634 | -0.019 | 0.195 | 0.107 |  |
| **Grazing ~ Hard Rock * Site** | | | | | | | |
| (Intercept) | 0.468 | 0.111 | 4.204 | 0.246 | 0.691 | <0.001 | *** |
| ROCK | -0.029 | 0.012 | -2.324 | -0.054 | -0.004 | 0.023 | * |
| Site 2 | 0.136 | 0.153 | 0.887 | -0.169 | 0.440 | 0.378 |  |
| Site 3 | 0.468 | 0.155 | 3.021 | 0.159 | 0.777 | 0.004 | ** |
| Site 4 | 0.198 | 0.150 | 1.322 | -0.101 | 0.497 | 0.191 |  |
| ROCK: Site 2 | -0.048 | 0.039 | -1.228 | -0.127 | 0.030 | 0.224 |  |
| ROCK: Site 3 | 0.036 | 0.035 | 1.026 | -0.034 | 0.106 | 0.308 |  |
| ROCK: Site 4 | -0.131 | 0.106 | -1.239 | -0.342 | 0.080 | 0.219 |  |
| **Grazing ~ *Acropora* digitate * Site** | | | | | | | |
| (Intercept) | 0.253 | 0.122 | 2.072 | 0.010 | 0.497 | 0.042 | * |
| ACD | 0.015 | 0.017 | 0.858 | -0.020 | 0.049 | 0.394 |  |
| Site 2 | 0.258 | 0.187 | 1.385 | -0.114 | 0.630 | 0.170 |  |
| Site 3 | 0.742 | 0.175 | 4.246 | 0.393 | 1.090 | <0.001 | *** |
| Site 4 | 0.211 | 0.192 | 1.095 | -0.173 | 0.594 | 0.277 |  |
| ACD: Site 2 | 0.000 | 0.017 | 0.005 | -0.035 | 0.035 | 0.996 |  |
| ACD: Site 3 | -0.010 | 0.023 | -0.444 | -0.057 | 0.036 | 0.658 |  |
| ACD: Site 4 | -0.028 | 0.018 | -1.586 | -0.063 | 0.007 | 0.117 |  |
| **Grazing ~ Turf Algae * Site** | | | | | | | |
| (Intercept) | 0.474 | 0.251 | 1.887 | -0.027 | 0.974 | 0.063 | * |
| TAL | -0.004 | 0.006 | -0.665 | -0.016 | 0.008 | 0.508 |  |
| Site 2 | 0.524 | 0.320 | 1.637 | -0.114 | 1.161 | 0.106 |  |
| Site 3 | 0.549 | 0.361 | 1.520 | -0.171 | 1.269 | 0.133 |  |
| Site 4 | -0.204 | 0.332 | -0.615 | -0.867 | 0.458 | 0.540 |  |
| TAL: Site 2 | -0.012 | 0.008 | -1.460 | -0.029 | 0.004 | 0.149 |  |
| TAL: Site 3 | 0.002 | 0.008 | 0.236 | -0.015 | 0.019 | 0.814 |  |
| TAL: Site 4 | 0.013 | 0.008 | 1.706 | -0.002 | 0.029 | 0.093 | . |
| **Grazing ~ Coralline algae * Site** | | | | | | | |
| (Intercept) | 0.099 | 0.199 | 0.499 | -0.298 | 0.497 | 0.619 |  |
| CCA | 0.007 | 0.006 | 1.247 | -0.004 | 0.018 | 0.216 |  |
| Site 2 | 0.523 | 0.269 | 1.945 | -0.013 | 1.058 | 0.056 | . |
| Site 3 | 0.792 | 0.328 | 2.419 | 0.139 | 1.446 | 0.018 | * |
| Site 4 | 0.921 | 0.306 | 3.012 | 0.311 | 1.531 | 0.004 | ** |
| CCA: Site 2 | -0.010 | 0.007 | -1.369 | -0.024 | 0.005 | 0.175 |  |
| CCA: Site 3 | -0.005 | 0.009 | -0.630 | -0.023 | 0.012 | 0.531 |  |
| CCA: Site 4 | -0.018 | 0.008 | -2.209 | -0.034 | -0.002 | 0.030 | * |
| **Planned Comparisons** | | | | | | | |
|  |  | **site** | **estimate** | **etd.err** | **t.value** | **p.value** | **signif.code** |
| **Spat Grazing ~ Cyano * Site** | | S2 – S1 | -0.317 | 0.169 | -1.874 | 0.226 |  |
|  |  | S3 – S1 | 0.495 | 0.153 | 3.227 | 0.008 | ** |
|  |  | S4 – S1 | -0.025 | 0.154 | -0.164 | 0.998 |  |
|  |  | S3 – S2 | 0.812 | 0.166 | 4.879 | <0.001 | *** |
|  |  | S4 – S2 | -0.520 | 0.151 | -3.439 | 0.005 | ** |
| **Spat Grazing ~ Hard Rock * Site** | | S2 – S1 | -0.340 | 0.152 | -2.239 | 0.108 |  |
|  |  | S3 – S1 | 0.358 | 0.154 | 2.321 | 0.090 | . |
|  |  | S4 – S1 | -0.087 | 0.149 | -0.583 | 0.927 |  |
|  |  | S3 – S2 | 0.698 | 0.149 | 4.677 | <0.001 | *** |
|  |  | S4 – S2 | -0.444 | 0.146 | -3.038 | 0.015 | * |
| **Spat Grazing ~ *Acropora* dig. * Site** | | S2 – S1 | -0.524 | 0.168 | -3.113 | 0.012 | * |
|  |  | S3 – S1 | 0.398 | 0.158 | 2.527 | 0.057 | . |
|  |  | S4 – S1 | -0.353 | 0.173 | -2.033 | 0.168 |  |
|  |  | S3 – S2 | 0.921 | 0.170 | 5.428 | <0.001 | *** |
|  |  | S4 – S2 | -0.751 | 0.175 | -4.289 | <0.001 | *** |
| **Frag Grazing ~ Cyano * Site** | | S2 – S1 | 0.067 | 0.173 | 0.388 | 0.977 |  |
|  |  | S3 – S1 | 0.551 | 0.157 | 3.514 | 0.004 | ** |
|  |  | S4 – S1 | 0.189 | 0.158 | 1.196 | 0.603 |  |
|  |  | S3 – S2 | 0.484 | 0.170 | 2.844 | 0.025 | * |
|  |  | S4 – S2 | -0.362 | 0.155 | -2.343 | 0.087 | . |
| **Frag Grazing ~ Hard Rock * Site** | | S2 – S1 | 0.136 | 0.153 | 0.887 | 0.789 |  |
|  |  | S3 – S1 | 0.468 | 0.155 | 3.021 | 0.015 | * |
|  |  | S4 – S1 | 0.198 | 0.150 | 1.322 | 0.519 |  |
|  |  | S3 – S2 | 0.332 | 0.150 | 2.214 | 0.114 |  |
|  |  | S4 – S2 | -0.270 | 0.147 | -1.833 | 0.241 |  |
| **Frag Grazing ~ *Acropora* dig. * Site** | | S2 – S1 | 0.258 | 0.187 | 1.385 | 0.485 |  |
|  |  | S3 – S1 | 0.742 | 0.175 | 4.246 | <0.001 | *** |
|  |  | S4 – S1 | 0.211 | 0.192 | 1.095 | 0.670 |  |
|  |  | S3 – S2 | 0.483 | 0.188 | 2.567 | 0.052 | . |
|  |  | S4 – S2 | -0.531 | 0.194 | -2.736 | 0.034 | * |
| **Frag Grazing ~ CCA * Site** | | S2 – S1 | 0.523 | 0.269 | 1.945 | 0.195 |  |
|  |  | S3 – S1 | 0.792 | 0.328 | 2.419 | 0.072 | . |
|  |  | S4 – S1 | 0.921 | 0.306 | 3.012 | 0.016 | * |
|  |  | S3 – S2 | 0.270 | 0.316 | 0.853 | 0.806 |  |
|  |  | S4 – S2 | 0.128 | 0.348 | 0.369 | 0.980 |  |


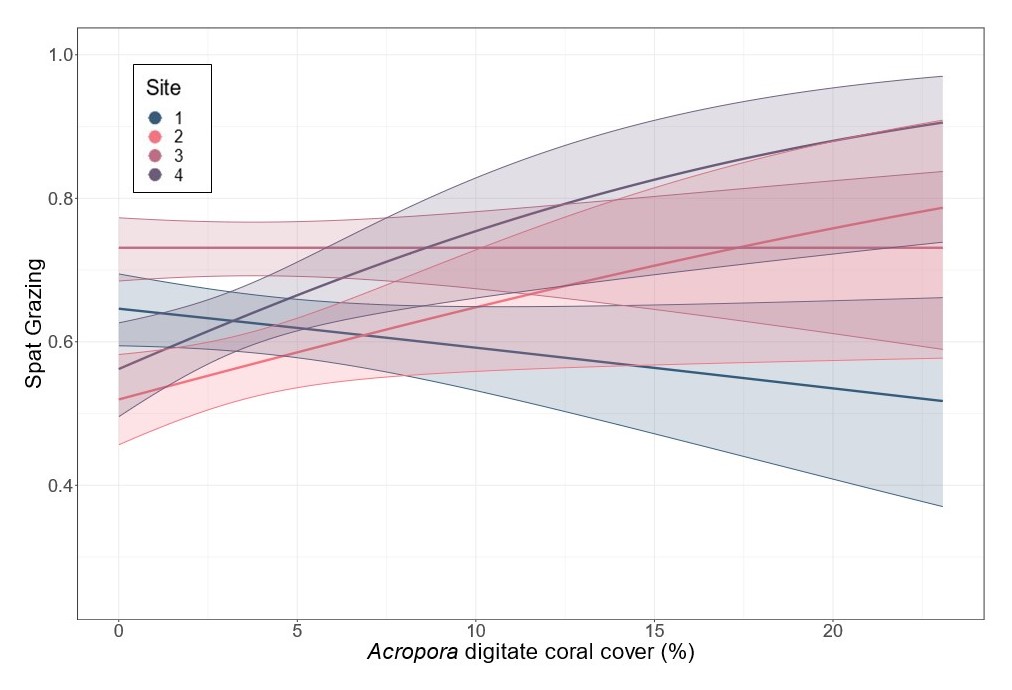


**Figure S1**. Logistic relationship between the percent cover of *Acropora* digitate corals (including corymbose morphology) and grazing of coral spat at the 2-d timepoint. The data was recorded from device plots (0.25 m^2^, n=31-41) and separated by site (n=4).


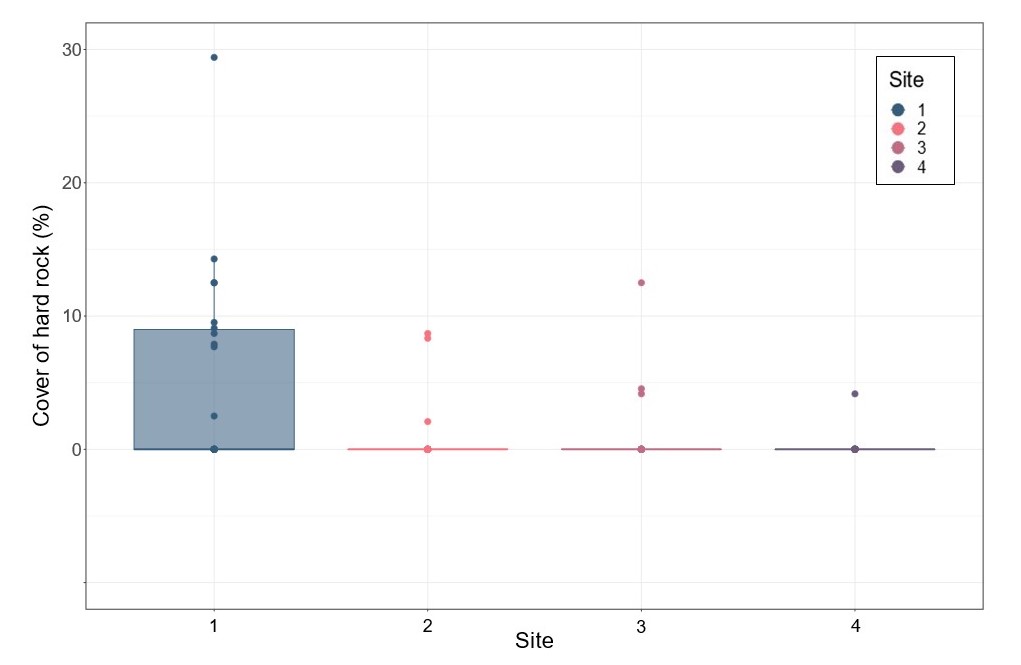


**Figure S2**. Percent cover of hard, bare rock at the 2-d timepoint and recorded in device plots (0.25 m^2^). The data was separated by site (n=4) and annotated by plot (n=31-41).

**2.5. Waterflow and sedimentation**

**Table S10.** Model output for environmental data, water flow and sedimentation. Linear models (lm; R Software) were used data values (water flow or sedimentation) or PC1 as the response, with site or pod type (Sed or Turf) as the predictors. Models are defined in the table. Significance codes represent p values, with “*” as <0.05, “**” as <0.01, and “***” as <0.001.

| **Environmental Models** | | | | | | | |
| --- | --- | --- | --- | --- | --- | --- | --- |
| **Water flow** | | | | | | | |
| **Linear Models** | |  |  |  |  |  |  |
| *Diffusion Factor* | |  |  |  |  |  |  |
| **DF ~ Site** | **Estimate** | **Std.Error** | **Statistic** | **Cf.Low** | **Cf.High** | **p.value** | **Significant Code** |
| (Intercept) | 4.803 | 0.208 | 23.109 | 4.362 | 5.243 | 0.000 | *** |
| Site2 | -0.461 | 0.294 | -1.568 | -1.084 | 0.162 | 0.136 |  |
| Site3 | -0.252 | 0.294 | -0.856 | -0.875 | 0.371 | 0.405 |  |
| Site4 | -0.332 | 0.294 | -1.130 | -0.955 | 0.291 | 0.275 |  |
| Residual standard error: 0.4647 on 16 degrees of freedom | | | |  |  |  |  |
| Multiple R-squared: 0.1407, Adjusted R-squared: -0.02036 | | | | |  |  |  |
| F-statistic: 0.8736 on 3 and 16 DF, p-value: 0.4753 | | | |  |  |  |  |
| *Dissolution Rate* | |  |  |  |  |  |  |
| **DR ~ Site** | **Estimate** | **Std.Error** | **Statistic** | **Cf.Low** | **Cf.High** | **p.value** | **Significant Code** |
| (Intercept) | 23.314 | 1.039 | 22.435 | 21.111 | 25.517 | 0.000 | *** |
| Site 2 | -2.304 | 1.470 | -1.568 | -5.420 | 0.811 | 0.136 |  |
| Site 3 | -1.258 | 1.470 | -0.856 | -4.374 | 1.857 | 0.405 |  |
| Site 4 | -1.661 | 1.470 | -1.130 | -4.776 | 1.455 | 0.275 |  |
| Residual standard error: 2.324 on 16 degrees of freedom | | | |  |  |  |  |
| Multiple R-squared: 0.1407, Adjusted R-squared: -0.02036 | | | | |  |  |  |
| F-statistic: 0.8736 on 3 and 16 DF, p-value: 0.4753 | | | |  |  |  |  |
| **Planned Comparisons** | |  |  |  |  |  |  |
| **Site** | | **estimate** | **std.err** | **t.value** | **p.value** |  |  |
| S2 - S3 | | -0.209 | 0.294 | -0.712 | 0.487 |  |  |
| S2 - S4 | | -0.129 | 0.294 | -0.438 | 0.667 |  |  |
| S3 - S4 | | 0.081 | 0.294 | 0.274 | 0.788 |  |  |
| **Sedimentation** | | | | | | | |
| **Linear Models** | |  |  |  |  |  |  |
| *Principal Component* | |  |  |  |  |  |  |
| **PC1 ~ Site** | | **estimate** | **std.err** | **t.value** | **p.value** |  |  |
| (Intercept) | | -0.09686 | 0.26219 | -0.369 | 0.717 |  |  |
| Site 2 | | 0.0605 | 0.37079 | 0.163 | 0.872 |  |  |
| Site 3 | | -0.07465 | 0.37079 | -0.201 | 0.843 |  |  |
| Site 4 | | 0.40158 | 0.37079 | 1.083 | 0.295 |  |  |
| Residual standard error: 0.5863 on 16 degrees of freedom | | | |  |  |  |  |
| Multiple R-squared: 0.1079, Adjusted R-squared: -0.05942 | | | | |  |  |  |
| F-statistic: 0.6448 on 3 and 16 DF, p-value: 0.5974 | | | |  |  |  |  |
|  |  |  |  |  |  |  |  |
| **PC1 ~ Sed Pod** | | **estimate** | **std.err** | **t.value** | **p.value** | **signif.code** |  |
| (Intercept) | | -0.7955 | 0.1773 | -4.487 | 0.000285 | *** |  |
| Sed_Pod | | 7470.503 | 1467.441 | 5.091 | 7.63E-05 | *** |  |
| Residual standard error: 0.3747 on 18 degrees of freedom | | | |  |  |  |  |
| Multiple R-squared: 0.5901, Adjusted R-squared: 0.5674 | | | | |  |  |  |
| F-statistic: 25.92 on 1 and 18 DF, p-value: 7.63e-05 | | | |  |  |  |  |
|  |  |  |  |  |  |  |  |
| **PC1 ~ Turf Pod** | | **estimate** | **std.err** | **t.value** | **p.value** | **signif.code** |  |
| (Intercept) | | 1.125 | 0.2363 | 4.76 | 0.000156 | *** |  |
| Turf_Pod | | -1033.58 | 203.027 | -5.091 | 7.6E-05 | *** |  |
| Residual standard error: 0.3747 on 18 degrees of freedom | | | |  |  |  |  |
| Multiple R-squared: 0.5901, Adjusted R-squared: 0.5674 | | | | |  |  |  |
| F-statistic: 25.92 on 1 and 18 DF, p-value: 7.63e-05 | | | |  |  |  |  |
|  |  |  |  |  |  |  |  |
| *In situ deposition* | |  |  |  |  |  |  |
| **Sediment ~ Site** | **estimate** | **std.err** | **statistic** | **cf.low** | **cf.high** | **p.value** | **signif.code** |
| (Intercept) | 0.00048 | 0.00019 | 2.519 | 0.00009 | 0.00086 | 0.016 | * |
| Site 2 | 0.00022 | 0.00027 | 0.814 | -0.00032 | 0.00076 | 0.421 |  |
| Site 3 | 0.00018 | 0.00027 | 0.664 | -0.00036 | 0.00072 | 0.511 |  |
| Site 4 | 0.00009 | 0.00027 | 0.347 | -0.00045 | 0.00063 | 0.731 |  |
| Residual standard error: 5.451e-05 on 16 degrees of freedom | | | | |  |  |  |
| Multiple R-squared: 0.2708, Adjusted R-squared: 0.134 | | | |  |  |  |  |
| F-statistic: 1.98 on 3 and 16 DF, p-value: 0.1576 | | | |  |  |  |  |
|  |  |  |  |  |  |  |  |
| **Sed Pod ~ Site** | **estimate** | **std.err** | **statistic** | **cf.low** | **cf.high** | **p.value** | **signif.code** |
| (Intercept) | 6.34E-05 | 2.44E-05 | 2.603 | 1.18E-05 | 0.0001151 | 0.019 | * |
| Site 2 | 6.12E-05 | 3.45E-05 | 1.776 | -1.19E-05 | 0.0001343 | 0.095 |  |
| Site 3 | 3.27E-05 | 3.45E-05 | 0.949 | -4.04E-05 | 0.0001058 | 0.357 |  |
| Site 4 | 7.83E-05 | 3.45E-05 | 2.270 | 5.18E-06 | 0.0001513 | 0.037 | * |
| Residual standard error: 5.451e-05 on 16 degrees of freedom | | | | |  |  |  |
| Multiple R-squared: 0.2708, Adjusted R-squared: 0.134 | | | |  |  |  |  |
| F-statistic: 1.98 on 3 and 16 DF, p-value: 0.1576 | | | |  |  |  |  |
|  |  |  |  |  |  |  |  |
| **Turf Pod ~ Site** | **estimate** | **std.err** | **statistic** | **cf.low** | **cf.high** | **p.value** | **signif.code** |
| (Intercept) | 0.00089 | 0.00019 | 4.634 | 0.00048 | 0.00129 | 0.0003 | *** |
| Site 2 | 0.00037 | 0.00027 | 1.378 | -0.00020 | 0.00095 | 0.187 |  |
| Site 3 | 0.00032 | 0.00027 | 1.187 | -0.00025 | 0.00090 | 0.252 |  |
| Site 4 | 0.00011 | 0.00027 | 0.395 | -0.00047 | 0.00068 | 0.698 |  |
| Residual standard error: 0.0004285 on 16 degrees of freedom | | | | |  |  |  |
| Multiple R-squared: 0.1373, Adjusted R-squared: -0.02441 | | | | |  |  |  |
| F-statistic: 0.8491 on 3 and 16 DF, p-value: 0.4872 | | | |  |  |  |  |
| **Planned Comparisons** | |  |  |  |  |  |  |
| **site** | | **pod type** | **estimate** | **std.err** | **t.value** | **p.value** |  |
| S2 - S3 | | Sed | -2.85E-05 | 3.45E-05 | -0.827 | 0.421 |  |
|  |  | Turf | -5.16E-05 | 2.71E-04 | -0.191 | 0.851 |  |
| S2 - S4 | | Sed | 1.70E-05 | 3.45E-05 | 0.494 | 0.628 |  |
|  |  | Turf | -0.000266 | 0.000271 | -0.983 | 0.34 |  |
| S3 - S4 | | Sed | 4.55E-05 | 3.45E-05 | 1.321 | 0.205 |  |
|  |  | Turf | -0.000215 | 0.000271 | -0.792 | 0.44 |  |


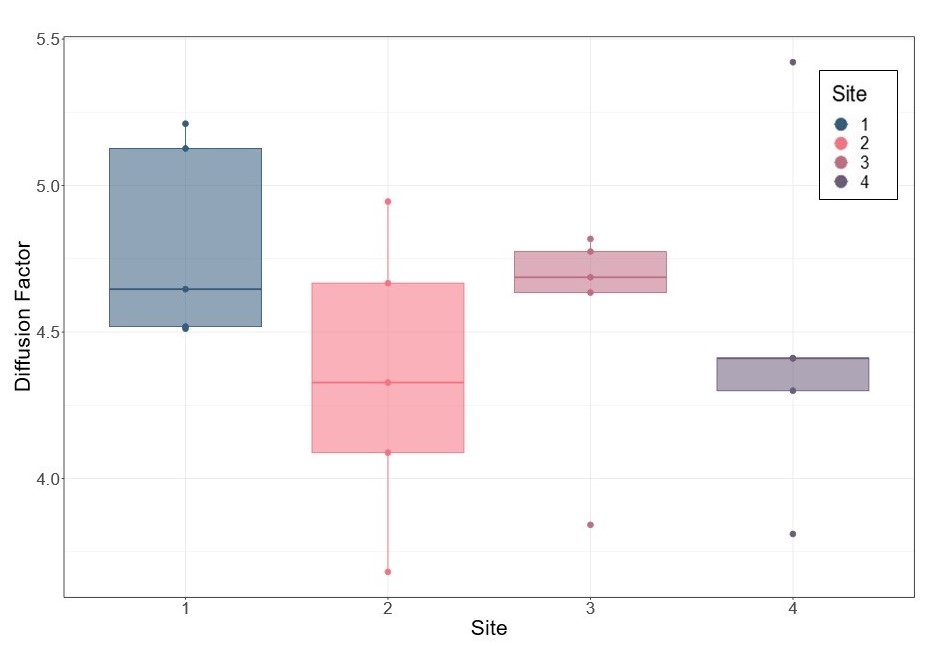


**Figure S3**. Water flow displayed as Diffusion Factor across sites. The values were obtained from the deployment of clod cards (n=5) at one timepoint for each experimental site (n=4).
